# Precision Medicine in Plant Food Allergy: a Systematic Review of Biomarkers Under a Clinical Approach

**DOI:** 10.1007/s12016-026-09136-8

**Published:** 2026-02-25

**Authors:** M. L. Somoza, E. Nuñez-Borque, I. M. Sánchez-Guerrero, O. Uriel, E. Marchan, M. Belver, A. Ruiz-Sánchez, R. Jiménez-Saiz, M. J. Goikoetxea

**Affiliations:** 1 Department of Allergy, Hospital Universitario Infanta Leonor, Madrid, Spain; 2 Department of Immunology, Instituto de Investigación Sanitaria Hospital Universitario La Princesa (IIS-Princesa), Universidad Autónoma de Madrid (UAM), Madrid, Spain; 3 Faculty of Medicine, Universidad Alfonso X El Sabio (UAX), Villanueva de la Cañada, Madrid, Spain; 4 Department of Allergy, Hospital Clínico Universitario Virgen de la Arrixaca, Murcia, Spain; 5 Department of Allergy, Hospital Universitario Araba, Vitoria-Gasteiz, Spain; 6 Department of Allergy, Complejo Hospitalario Universitario de Toledo, Toledo, Spain; 7 Department of Allergy, Hospital Universitario de La Princesa, IIS-Princesa, Madrid, Spain; 8 Faculty of Experimental Sciences, Universidad Francisco de Vitoria (UFV), Madrid, Spain; 9 Department of Medicine, McMaster Immunology Research Centre (MIRC), Schroeder Allergy and Immunology Research Institute (SAIRI), McMaster University, Hamilton, ON Canada; 10 Department of Allergy and Clinical Immunology, Clínica Universidad de Navarra, Pamplona, Spain; 11 Health Research Institute, Instituto de Investigación Sanitaria de Navarra (IdisNA), Pamplona, Spain

**Keywords:** Biomarker, Component revolved diagnosis, IgE, Plant food allergy, Precision medicine

## Abstract

Plant food allergy (FA) is a major global health problem, being peanut one of the most studied allergenic foods worldwide. Biomarkers (Bms) are essential tools for precision medicine, guiding diagnosis, risk stratification, and treatment. However, evidence on their clinical applicability in plant FA remains limited and fragmented. Therefore, we aim to systematically review the Bms identified in plant FA related to several aspects of the pathology. This systematic review was performed in accordance with the PRISMA guidelines. A comprehensive search was conducted in three databases (*PubMed*, *Web of Sciences* and *Cochrane Library*) between July 2019 and July 2024. Articles were screened using predefined inclusion and exclusion criteria, and categorized into five thematic sections: sensitization, tolerance, threshold, severity and follow-up treatment. Risk-of-bias and certainty of evidence were assessed using validated tools and the GRADE approach. From the 733 articles found, 71 studies met inclusion criteria and were selected for data extraction. A majority of them were high-quality publications and predominantly involved pediatric cohorts from Europe and North America. Peanut was the most studied food, followed by nuts and wheat, while fruits and seeds were rarely represented. Across all sections, most of the studies were related to severity and tolerance. Specific IgE to whole extracts or molecular components were the most frequent Bms, followed by skin prick test. Emerging Bms were identified, although they remain in early validation stages. Risk-of-bias and certainty of evidence were primarily moderate, reflecting the exploratory nature of most of the included studies. This review highlights the predominance of classical Bms and the promise of novel candidates for future clinical integration. However, research remains allergen-, age- and region-restricted, underscoring the need for multicenter studies, representative patient samples, and validation of emerging Bms to achieve globally applicable precision medicine in plant FA.

**Supplementary Information:**

The online version contains supplementary material available at 10.1007/s12016-026-09136-8.

## Introduction

Precision medicine is an evolving strategy that incorporates individualized assessments for disease prevention and personalized treatment [1]. The application of biomarkers (Bms) to clinical practice allows patients to be stratified into different risk levels, identifying how to manage them more optimally. Currently, significant progress has been made in characterizing Bms to define asthma endotypes, prompted by the increasing availability of biologics [2]. However, evidence about precision medicine in food allergy (FA) is scarce, focusing mainly on the triggers of FA development, severity, tolerance or immunotherapy response [3].

Plant FA is a main problem world-wide, and specially peanut allergy due to its high prevalence, its increasing incidence and its association with severe reactions [4, 5]. Beyond peanuts, there are other relevant plant-food allergens showing high prevalence in some regions, such as fruits, vegetables, legumes and cereals [6, 7]. Clinical guidelines recommend using some Bms, such as specific IgE (sIgE) to Ara h 2, Cor a 14 or Ana o 3, to predict tolerance based on extensive research performed in plant-food allergic patients frequently sensitized to storage proteins [8, 9]. However, other patterns have been described across the world: from the typically mild and localized pollen allergy syndrome, to the heterogenic lipid transfer protein allergy, which can manifest from mild to severe symptoms, very often unpredictably and conditioned by cofactors [10, 11]. In this scenario, the predictors of tolerance and severity are related to the patient’s sensitization profile and, therefore, could be heterogeneous across different populations.

The component-resolved diagnosis (CRD) in FA contributes to endotype patients by identifying specific molecular sensitization patterns linked to different clinical expressions, discriminating between primary allergies and cross-reactivity syndromes [12]. Nevertheless, CRD has limitations when it comes to comprehensively addressing the responses for FA patients, as variability has been observed between populations in the serotypes of different patient cohorts [13]. Moreover, the development of CRD has mainly focused on a restricted panel of food allergens [14]. It is important to note that the identification of new Bms does not always imply their direct clinical implementation worldwide due to economic and logistic barriers. Similarly, this also applies to other diagnostic approaches, as well as basophil activation test (BAT) [15] or allergic mediators (interleukins) and cells (mainly B and CD4 T cells) [16, 17], which may require costly equipment and highly trained personnel.

Even more, new drugs and approaches are emerging for plant FA management, which requires adaptation to each patient. Therefore, Bms of indication, treatment monitoring and efficacy must be characterized. Once again, efforts are mainly focused on peanut allergy, hoping this path can serve as a guide for other FA treatments [18]. However, other plant food-based therapies are still far from those developed against peanut allergy [19, 20].

Beyond the main challenges of precision medicine in FA, and focusing specifically on the needs of clinical practice, there are other aspects—in addition to predicting severity, tolerance, or treatment monitoring—that must be considered to fully characterize the diagnosis of allergic patients to plant foods. To perform a real precision medicine in daily clinical practice, Bms should provide information on the following clinical questions: (i) how FA sensitization is developed; (ii) the prediction of the clinical relevance of sensitization; (iii) the determination of susceptibility to severe reactions or symptoms; (iv) the high/low or certain threshold at which reactions occur; and (v) the monitoring of responses to allergen immunotherapy or immunomodulatory drugs. Therefore, we aim to systematically review the Bms identified in plant FA related to these aspects of the pathology.

## Methods

### Search Strategy

This systematic review was performed in accordance with the PRISMA guidelines [21]. The selection of articles was limited to manuscripts published between July 2, 2019, and July 2, 2024. The search was carried out across three databases (*PubMed*, *Web of Science*, and *Cochrane Library*) and structured in five thematic sections using a common set of terms, combined with keywords specific to each section: “sensitization”, “tolerance”, “threshold”, “severity” and “follow-up treatment” (Supplementary Table 1).

### Inclusion and Exclusion Criteria

All studies included in this review were selected based on a set of general inclusion and exclusion criteria, applicable to all sections. Eligible studies (including research articles, short communications, or letters to the editor) were required to present original human data, be written in English, identify Bms, and show statistically significant association with plant FA. Exclusion criteria were studies on eosinophilic esophagitis; allergies to non-plant food allergens; clinical investigations that did not involve Bm identification; publications in languages other than English; case reports, conference abstracts, preprints, or review articles; and studies conducted in animal models or human ex vivo material (cells or tissues).

In addition to these general considerations, the five thematic sections were assessed using the following specific eligibility criteria:

For the “sensitization” section, studies about Bm predicting plant food-sensitization acquisition were included. These involve patients with confirmed sensitization regardless of whether oral food challenge (OFC) tests were performed. Controls were required to be non-sensitized individuals. Studies comparing allergic *vs* tolerant sensitized patients and those focusing on sensitization to specific allergens (i.e., Ara h 2 sIgE) were excluded because they do not directly reflect Bms of sensitization development.

For the “tolerance” section, articles about prediction of clinical reactivity were considered. Only studies involving plant food-allergic patients with diagnosis confirmed through medically supervised OFC (open-label, single- or double-blind) were included, excluding longitudinal prediction of future tolerance. The control group did not apply in this section, since all the studies compared subjects with positive *vs* negative OFC.

For the “severity” section, studies regarding the susceptibility to present severe reactions were considered. These studies compare plant food-allergic individuals who had experienced systemic or anaphylactic reactions with those who had only local or mild symptoms. Articles focusing exclusively on acute-phase anaphylaxis or on populations composed solely of anaphylactic patients were excluded, as the focus was on identifying baseline Bms of susceptibility to severe reactions. Articles showing at least two groups of patients with different levels of severity were considered.

For the “threshold” section, studies on different threshold levels were included. Specifically, articles in which plant food-allergic patients were challenged under medical supervision with clearly reported subjective or objective threshold doses were considered. Studies that did not confirm allergy through OFC or that presented data collected after immunomodulatory treatment (i.e., immunotherapy) were excluded.

For the “follow-up treatment” section, studies about the response to treatments were included. Patients undergoing validated or non-validated treatments were considered: probiotics, biologic agents, epicutaneous immunotherapy (EPIT) or oral immunotherapy (OIT). Participants had to be characterized at baseline to allow for the evaluation of associations between parameters and treatment efficacy. The definition of efficacy was interpreted broadly and could include desensitization, even without assessment of sustained unresponsiveness.

### Analysis of the Articles Included in the Review

All publications retrieved through the various search strategies were exported. Duplicates across the three databases and the five thematic sections were identified and removed. Subsequently, two authors (ENB and RJS) independently screened the titles and abstracts and discarded those that did not fulfill the inclusion criteria. Discrepancies were resolved by a third reviewer (MJG).

Once the final list of included studies was established, two additional reviewers (MJG and MLS) independently re-evaluated the titles and abstracts to reassign each article to the appropriate thematic section. The full text of each article was then independently peer-reviewed to confirm its inclusion in the systematic review. Specifically, the “sensitization” section was reviewed by MLS and MB; the “tolerance” section by OUV and IMSG; the “threshold” section by MB and MJG; the “severity” section by MJG, MLS and EM; and the “follow-up treatment” section by EM and MLS. All the reviewers of the final list were practicing allergists to keep the clinical perspective of the analysis.

For each included article, the following data were extracted: journal (position in its area of knowledge, impact factor, and subject area), year of publication, country, pathology studied (condition, physiological state or associated therapeutic intervention), characteristics of the study population (mean age, sample size and control group), description of the Bm (name, type—predictive, follow-up, treatment response, prognostic or susceptibility—, detection technique, and units of measure), and evidence of association (positive or inverse).

### Biomarker Analysis

Bms were considered when the same measurable item was similarly evaluated in the identical food allergen. For the analysis of specific peptides, when ≥ 3 epitopes were measured using the same assay (i.e., epitope microarray) the panel was considered as a single Bm according to their association. Models studying more than one different Bm together were not considered. Concerning skin prick test (SPT), it was included as a Bm only if the authors described the technique and positive criteria used in the methods section. The type of Bm was considered following FDA-NIH Bm working group classification [22].

Bms were included when a significant association with FA was observed. Association was defined as positive when the presence of allergy, higher eliciting dose, or severity were associated with the Bm; and inverse when the absence of allergy (tolerance), lower eliciting doses, or severity grade were associated with the Bm. Regarding the sensitization and follow-up treatment sections, a positive association was defined as an increase in Bm levels (compared to non-sensitized group, baseline or progressively along procedures) related to a good intervention efficacy, while an inverse association was a decrease in Bm levels related to sensitization or good intervention efficacy.

### Risk-of-bias and Certainty Assessment

The risk-of-bias and the certainty of the articles were measured using a hybrid strategy combining artificial intelligence (AI) tools (conducted by ENB and RJS) with human evaluations from the reviewers (MJG, MLS, OUV and IMSG). For this purpose, an automated process based on a large-scale language model (OpenAI GPT-4) was implemented [23], which extracted the relevant methodological information directly from the article text. This approach ensures an objective initial extraction of parameters, reducing human bias and improving the transparency and reproducibility of quality assessment.

The risk-of-bias assessment was performed by programming the AI to classify each article according to its design. After this, a risk-of-bias tool was applied according to the type of study identified [24, 25]: Risk-Of-Bias In Non-randomized Studies of Interventions (ROBINS-I) for non-randomized studies; Appraisal tool for Cross-Sectional Studies (AXIS) for cross-sectional studies; Quality Assessment of Diagnostic Accuracy Studies (QUADAS-2) for studies of diagnostic accuracy; and Cochrane tool for assessing risk-of-bias in randomized trials (Rob2) for randomized trials. Tables were generated for each article with detailed assessments and an overall evaluation of the risk of bias, always based on the data explicitly reported in the publications.

The Grading of Recommendations Assessment, Development and Evaluation (GRADE) system was used to assess the certainty of the evidence [26]. For each article, the following aspects were considered: risk of bias, inconsistency, imprecision, indirectness and publication bias, as well as a general classification of certainty.

All the evaluations performed by the AI were subjected to peer review by MJG, MLS, OUV and IMSG; and any discrepancies were resolved by consensus, to ensure the coherence, transparency and methodological soundness of the process.

## Results

**Fig. 1:**
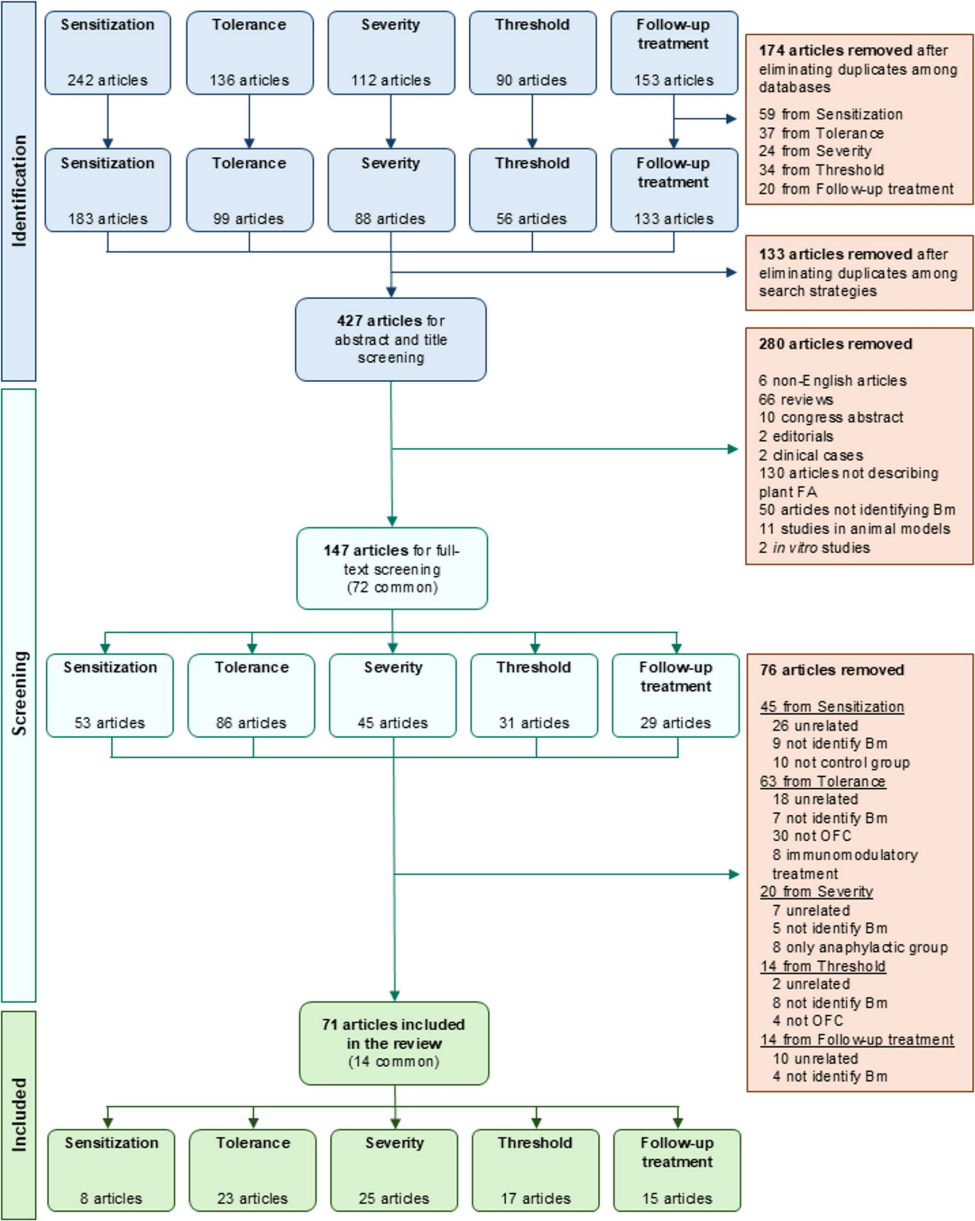
Study selection process. From an initial pool of 733 articles, duplicates were removed, resulting in 427 unique references. After peer review of titles and abstracts, 147 articles were selected, with 72 of them overlapping across multiple thematic sections. Following full-text review, a total of 71 studies were included for data extraction, with 14 of them overlapping among different thematic sections (11 commons between 2 sections, and 3 commons among 3 sections). Biomarker, Bm; Food allergy, FA; Oral food challenge, OFC

The initial search identified a total of 733 studies. After removing duplicates across the various databases and search strategies, 427 unique articles were retained. Titles and abstracts were peer-reviewed, leading to the exclusion of 280 studies. At this stage, the main reasons for exclusion were the type of publication (e.g., reviews, case reports or editorials), unrelatedness to plant FA, and absence of Bm identification. The remaining 147 articles were assigned to the five predefined thematic sections, and their full texts were peer-reviewed. During this in-depth review, 76 additional articles were excluded, mainly due to the absence of Bm identification, lack of alignment with the objective of the respective thematic section, or issues related to the control group when applied (e.g., absence of OFC or control group, inclusion of patients undergoing immunotherapy, or inclusion of only anaphylactic cases). Ultimately, 71 articles were selected for data extraction **(**Fig. 1**)**.

### Characteristics of the Studies

**Fig. 2:**
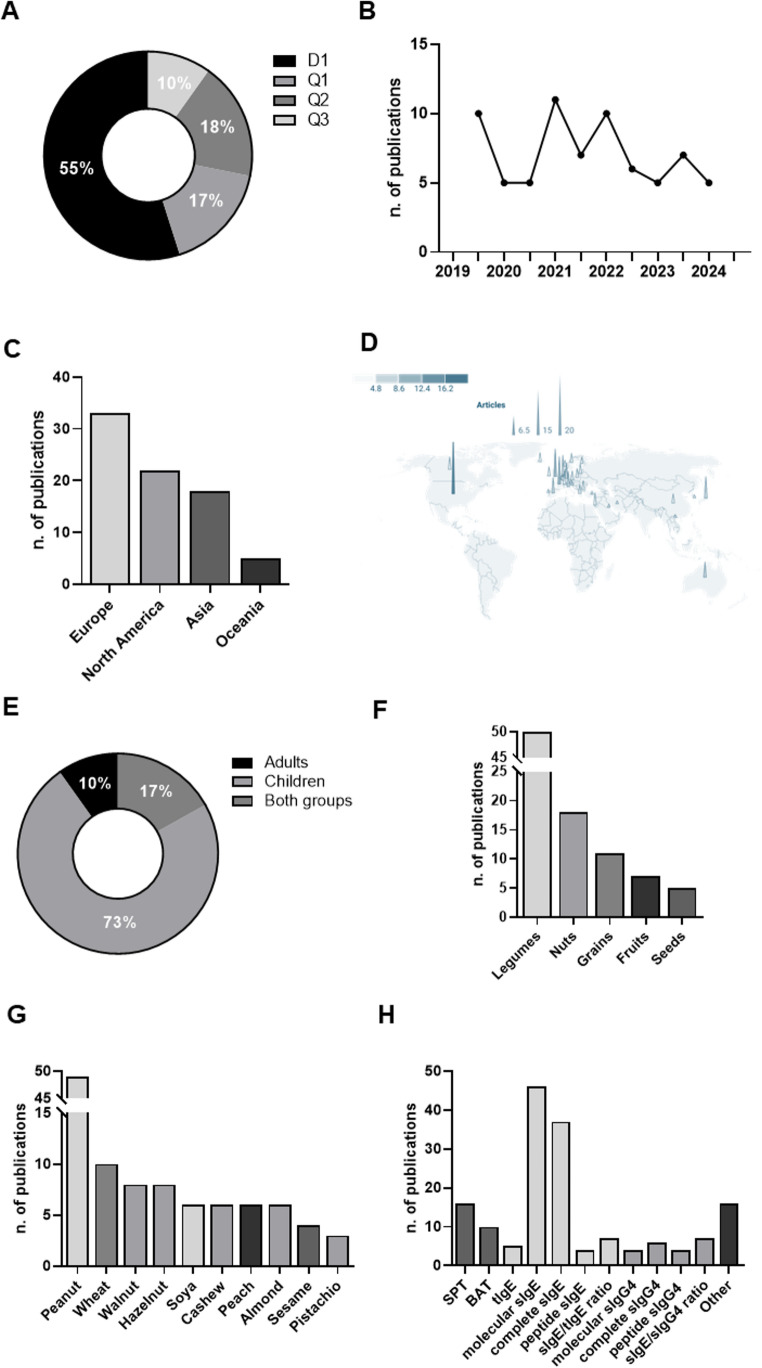
Characteristics of the 71 studies included in the review. (**A**) Distribution of articles by journal quartile. D1: first decile (top 10% of most cited journals in its category); Q1: first quartile (top 25%); Q2: second quartile (25 to 50%); Q3: third quartile (50% to 75%). (**B**) Number of articles published by six-month intervales, from July 2, 2019, to July 2, 2024. (**C**) Number of studies by continent of origin. Some articles included populations from multiple continents (*n* = 5). (**D**) Geographical distribution of studies by country. The size and color of each spot indicate the number of studies. Some studies included patients from multiple countries (*n* = 12). United States (*n* = 20); France (*n* = 9); United Kingdom (*n* = 9); Japan (*n* = 7); Netherlands (*n* = 7); Australia (*n* = 5); Germany (*n* = 5); Italy (*n* = 5); Spain (*n* = 5); Canada (*n* = 4); Israel (*n* = 4); Switzerland (*n* = 4); Bulgaria (*n* = 3); China (*n* = 3); Czech Republic (*n* = 3); Greece (*n* = 3); Iceland (*n* = 3); Ireland (*n* = 3); Lithuania (*n* = 3); Poland (*n* = 3); Sweden (*n* = 3); Finland (*n* = 2); Luxembourg (*n* = 2); Norway (*n* = 2); Austria (*n* = 1); Belgium (*n* = 1); Denmark (*n* = 1); Iran (*n* = 1); Kuwait (*n* = 1); Portugal (*n* = 1); Slovenia (*n* = 1); South Korea (*n* = 1); Turkey (*n* = 1); Vietnam (*n* = 1). Image created with *Datawrapped.com*. (**E**) Distribution of articles by the age group of the study population. Children are defined as individuals under 18 years old; adults are 18 years old or older. (**F**) Number of studies by plant food source. Some studies included multiple plant food sources (*n* = 10). Legumes: peanut and soya; Nuts: walnut, hazelnut, cashew, almond, and pistachio; Grains: wheat; Fruits: peach; Seeds: sesame. (**G**) Number of studies by specific plant food source. Some studies evaluated multiple sources (*n* = 13). (**H**) Number of studies by Bm type. Some studies assessed more than one Bm (*n* = 44). Skin prick test, SPT; basophil activation test, BAT; specific IgE, sIgE; total IgE, tIgE; specific IgG4, sIgG4

Among the 71 articles included, 72% (*n* = 51) were published in journals ranked in the first quartile of their respective categories (*Journal Citation Report*). Of these, 39 were in the first decile, reflecting a high level of quality and scientific relevance **(**Fig. 2A**)**. The overall mean impact factor of the included articles was 8.6. In terms of subject areas, most publications fell under the categories of *Allergy* (*n* = 64) and *Immunology* (*n* = 58), followed by *Pediatrics* (*n* = 5) and other categories (*n* = 5), such as *Nutrition & Dietetics*, *Food Science & Technology*, *Environmental Sciences*, and *Medicine*,* General & Internal*.

Despite the recognized importance of identifying effective Bms for plant FA, no consistent increase in the number of publications was observed between July 2019 and July 2024 **(**Fig. 2B**)**. Geographically, most studies were conducted in Europe, followed by North America, Asia, and Oceania. No studies were identified from South America, Africa or Antarctica **(**Fig. 2C**)**. The most represented countries were the United States of America (USA, *n* = 10), France, United Kingdom (*n* = 9), Japan, Netherlands (*n* = 7), Australia, Germany, Italy, and Spain (*n* = 5) **(**Fig. 2D**)**.

Most of the included studies were conducted in pediatric populations (*n* = 52). In contrast, only a small number of studies included both children and adults (*n* = 12), while a minority focused exclusively on adults (*n* = 7) **(**Fig. 2E**)**. In terms of the plant food groups evaluated, legumes (peanut and soy) were the most studied, followed by nuts (walnut, hazelnut, cashew, almond, and pistachio), grains (wheat), fruits (peach) and seeds (sesame) **(**Fig. 2F**)**. Considering specific foods, peanut was the most frequently investigated, followed, although to a lesser extent, by wheat, hazelnut and walnut **(**Fig. 2G**)**.

On the other hand, sIgE against both whole extracts and molecular components was the primary parameter analyzed across the included studies, underscoring the central role of this Bm in the diagnosis of FA. SPT and BAT were also widely used. Additionally, although less frequently, other Bms were assessed, such as sIgG4, peptide sIgE levels, and various ratios: sIgE to total IgE (tIgE) and sIgG4 to sIgE. Finally, a large and heterogeneous group of emerging Bms, still in the validation phase (*n* = 16), was identified: including proteins (*n* = 5), cellular platforms (*n* = 3), metabolites (*n* = 3), studies on other clinical parameters (*n* = 2), microbiota (*n* = 2), exhaled nitric oxide (FeNO, *n* = 1) and genetic factors (*n* = 1) **(**Fig. 2H**)**.

### Sensitization

Despite efforts in recent years to identify risk factors for food allergies and to implement preventive measures in the general population, the number of articles included in the sensitization section has been surprisingly low, as most of the studies evaluated the development of allergy rather than sensitization, and only few of them identified clear Bms. We included a total of eight articles about Bms predicting plant food-sensitization acquisition where a total of 12 Bms were identified from 7,279 subjects (Table 1). Four studies were performed in America (50%) [27–30], three in Europe (37.5%) [31–33] and one in Asia (12.5%) [34]. All of them, except one [30], included pediatric subjects, and most of the articles were cohorts analyzed at predetermined time periods (62.5%) [27, 29, 31–33].

**Table 1:** Studies about biomarker (Bm) predicting plant food-sensitization acquisition (*n* = 8). Italics* indicate the specific study population for that Bm within the total cohort. A positive association is related to an increase in the Bm according to the pathology/condition evaluated, while an inverse association is linked to a decrease in the Bm

	ARTICLE		STUDIED POPULATION						BIOMARKER				
Food	Reference	Year of publication	Country^1^	Sample size	Age	Range^2^	Control group	Pathology/Condition^3^	Name^4^	Type	Diagnostic technique (manufacturer)	Units^5^	Association
**Almond**	Tran et al. [34]	2024	Vietnam	110	25.9 months (mean)	13.1 (SD)	Yes	Food sensitization	SCH	Predictive	GPSkin Barrier Pro device (Gpower Inc.)	AU	Inverse
**Peanut**	Tsilochristou et al. [32]	2019	United Kingdom	681	NA	NA	Yes	Food sensitization	*S. aureus* colonization	Predictive	Bacteria culture	+/-	Positive
	Tran et al. [34]	2024	Vietnam	110	25.9 months (mean)	13.1 (SD)	Yes	Food sensitization	SCH	Predictive	GPSkin Barrier Pro device (Gpower Inc.)	AU	Inverse
	Goleva et al. [28]	2020	USA	84	11.5 years (mean)*	10.3–15.3 (SD)*	Yes	Food sensitization	PC1 proteins	Predictive	Proteomic analysis	ND	Positive
	Ran et al. [30]	2024	USA	229*	46 years (mean)	32–63 (IQR)	Yes	Food sensitization	urine metals: Lead (Pb)	Predictive	Mass spectrometry (ICP-MS)	LOD	Positive
**Peanut**,** walnut**,** soya**,** wheat**	Lee-Sarwar et al. [29]	2019	USA	779	NA	NA	Yes	Food sensitization	ω3 PUFAs	Predictive	Metabolon (Research Triangle Park)	ND	Inverse
									Total PUFAs	Predictive	Metabolon (Research Triangle Park)	ND	Inverse
									ω6 PUFAs	Predictive	Metabolon (Research Triangle Park)	ND	Inverse
**Peanut**,** wheat**	Tedner et al. [33]	2021	Norway, Sweden	2220	NA	NA	Yes	Food sensitization	sIgE	Predictive	ImmunoCAP (ThermoFisher)	kUA/L	Positive
**Peanut**,** wheat**,** soya**	Wärnberg Gerdin et al. [31]	2022	Norway, Sweden	2805	NA	NA	Yes	Food sensitization	TEWL	Predictive	Open-chamber DermaLab USB (Cortex)	g/m2/h	Positive
	Lee-Sarwar et al. [27]	2023	USA	371	NA	NA	Yes	Food sensitization	Fecal caffeine metabolite	Predictive	Metabolon (Research Triangle Park)	ND	Positive
									Fecal sphingolipid metabolites	Predictive	Metabolon (Research Triangle Park)	ND	Positive
									Fecal bilirubin metabolite	Predictive	Metabolon (Research Triangle Park)	ND	Inverse
**Wheat**,** soya**,** almond**,** walnut**,** hazelnut**,** cashew**,** sesame**	Goleva et al. [28]	2020	USA	84	11.5 years (mean)*	10.3–15.3 (SD)*	Yes	Food sensitization	PC1 proteins	Predictive	Proteomic analysis	ND	Positive

^1^*USA* United States of America. ^2^*IQR *Interquartile range, *SD* Standard deviation. ^4^*PUFAs* Polyunsaturated Fatty Acids, *PC1* Principal component 1, *sIgE* Specific IgE, *SCH* Stratum corneum hydration, *TEWL* Trans-epidermal water loss. ^5^*AU* Arbitrary units, *LOD* Limit of detection. *NA* Not applicable, *ND* Not determined

Most of the studies included atopic dermatitis subjects (50%) [28, 31, 32, 34], three of them studied diet/fecal characteristics (37.5%) [27, 29, 30] and one of them evaluated genetic factors (12.5%) [33]. Similarly, most of the studies analyzed multiple food allergen sensitization at the same time (62.5%) [27–29, 31, 33], while three articles were related exclusively to peanut (37.5%) [30, 32, 34] and one to almond (12.5%) [30].

### Tolerance

Tolerance was the second section with most articles, with 45 Bms identified from 23 studies based on 3,176 patients (Table 2). Eleven studies were conducted in Europe (47.8%) [35–45], seven in Asia (30.4%) [46–52], four in America (17.4%) [37, 53–55], two in Australia (8.7%) [56, 57] and three were transcontinental (13%) [36, 37, 54]. Specifically, the Netherlands was the most frequent country (17.4%) [35, 40, 41, 45], followed by the United Kingdom (13%) [36, 37, 39].

**Table 2:** Studies regarding prediction of clinical reactivity (*n* = 23). Italics* indicate the specific study population for that biomarker (Bm) within the total cohort. A positive association is related to an increase in the Bm according to the pathology/condition evaluated, while an inverse association is linked to a decrease in the Bm

	ARTICLE		STUDIED POPULATION						BIOMARKER				
Food	Reference	Year of publication	Country^1^	Sample size	Age	Range^2^	Control group	Pathology/Condition^3^	Name^4^	Type	Diagnostic technique (manufacturer)	Units^5^	Association
**Cashew**	Santos et al. [36]	2021	United Kingdom, Switzerland, Italy	92	5.1 years (median)	3–9 (IQR)	NA	Allergy (OFC +)	Cashew SPT	Predictive	Commercial extract (Stallergenes)	mm	Positive
									Cashew sIgE	Predictive	ImmunoCAP (ThermoFisher)	kUA/L	Positive
									Ana o 3 sIgE	Predictive	ImmunoCAP (ThermoFisher)	kUA/L	Positive
									Cashew BAT	Predictive	BAT (Flow-CAST)	%	Positive
	Cetinkaya et al. [46]	2021	Turkey	52*	61.18 months (median)*	42.21–104.53 (IQR)*	NA	Allergy (OFC +)	Cashew SPT	Predictive	SPT (Homemade extract)	mm	Positive
									Cashew sIgE	Predictive	ImmunoCAP (ThermoFisher)	kUA/L	Positive
									Cashew sIgE/tIgE	Predictive	ImmunoCAP (ThermoFisher)	NA	Positive
	Röntynen et al. [38]	2022	Finland	110	5.04 years (median)	1.41–16.66 (min-max)	NA	Allergy (OFC +)	Cashew SPT	Predictive	SPT (ALK extract)	mm	Positive
									Cashew sIgE	Predictive	ImmunoCAP (ThermoFisher)	kUA/L	Positive
									Ana o 3 sIgE	Predictive	ImmunoCAP (ThermoFisher)	kUA/L	Positive
									Cashew BAT	Predictive	BAT (Flow-CAST)	%	Positive
**Hazelnut**	Santos et al. [36]	2021	United Kingdom, Switzerland, Italy	92	5.1 years (median)	3–9 (IQR)	NA	Allergy (OFC +)	Hazelnut SPT	Predictive	Commercial extract (Stallergenes)	mm	Positive
									Hazelnut sIgE	Predictive	ImmunoCAP (ThermoFisher)	kU/L	Positive
									Cor a 1 sIgE	Predictive	ImmunoCAP (ThermoFisher)	kU/L	Positive
									Cor a 9 sIgE	Predictive	ImmunoCAP (ThermoFisher)	kU/L	Positive
									Cor a 14 sIgE	Predictive	ImmunoCAP (ThermoFisher)	kU/L	Positive
									Hazelnut BAT	Predictive	BAT (Flow-CAST)	%	Positive
	Duan et al. [54]	2021	Canada, Austria	107*	8.5 years (mean)	4.6–12.3 (IQR)	NA	Allergy (OFC +)	Cor a 14 sIgE	Predictive	ImmunoCAP (Thermo Fisher)	kU/L	Positive
									Hazelnut BAT	Predictive	BAT (Flow-CAST)	%	Positive
	Inoue et al. [47]	2020	Japan	91	7.3 years (median)	5.9–10.5 (IQR)	NA	Allergy (OFC +)	Alder sIgE	Predictive	ImmunoCAP (ThermoFisher)	kUA/L	Inverse
									Cor a 1 sIgE	Predictive	ImmunoCAP (ThermoFisher)	kUA/L	Inverse
									Cor a 9 sIgE	Predictive	ImmunoCAP (ThermoFisher)	kUA/L	Positive
**Peanut**	Ehlers et al. [35]	2021	Netherlands	12	35.6 years (mean)	27–63 (min-max)	NA	Allergy (OFC +)	VH3 family genes in peanut 2 S albumin-specific B cells	Predictive	Data sequence	%	Positive
									4 HCDR3 sequence motifs in peanut 2 S albumin-binding B cells	Predictive	Data sequence	%	Positive
									3 HCDR3 sequence motifs in peanut 2 S albumin-binding B cells	Predictive	Data sequence	%	Inverse
	Santos et al. [36]	2021	United Kingdom, Switzerland, Italy	92	5.1 years (median)	3–9 (IQR)	NA	Allergy (OFC +)	Peanut SPT	Predictive	Commercial extract (Stallergenes)	mm	Positive
									Peanut sIgE	Predictive	ImmunoCAP (ThermoFisher)	kUA/L	Positive
									Ara h 1 sIgE	Predictive	ImmunoCAP (ThermoFisher)	kUA/L	Positive
									Ara h 2 sIgE	Predictive	ImmunoCAP (ThermoFisher)	kUA/L	Positive
									Ara h 3 sIgE	Predictive	ImmunoCAP (ThermoFisher)	kUA/L	Positive
									Peanut BAT	Predictive	BAT (Flow-CAST)	%	Positive
									Ara h 1 BAT	Predictive	BAT (Flow-CAST)	%	Positive
									Ara h 2 BAT	Predictive	BAT (Flow-CAST)	%	Positive
									Ara h 6 BAT	Predictive	BAT (Flow-CAST)	%	Positive
	Santos et al. [37]	2020	United Kingdom, USA	981	ND	5–6 (min-max)	NA	Allergy (OFC +)	Peanut BAT	Predictive	BAT (Flow-CAST)	median	Positive
	Kaur et al. [56]	2021	Australia	222	8 years (median)	5–12 (IQR)	NA	Allergy (OFC +)	Ara h 1 sIgE	Predictive	ImmunoCAP (ThermoFisher)	kUA/L	Positive
									Ara h 2 sIgE	Predictive	ImmunoCAP (ThermoFisher)	kUA/L	Positive
									Ara h 6 sIgE	Predictive	ImmunoCAP (ThermoFisher)	kUA/L	Positive
									Peanut sIgE	Predictive	ImmunoCAP (ThermoFisher)	kUA/L	Positive
									Peanut SPT	Predictive	SPT (ND extract)	mm	Positive
	Lang et al. [53]	2022	USA	184	4 years (median)	2–7 (IQR)	NA	Allergy (OFC +)	Ara h 2 sIgE	Predictive	ImmunoCAP (ThermoFisher)	kUA/L	Positive
	Duan et al. [54]	2021	Canada, Austria	107*	8.5 years (mean)	4.6–12.3 (IQR)	NA	Allergy (OFC +)	Ara h 2 sIgE	Predictive	ImmunoCAP (Thermo Fisher)	kU/L	Positive
									Peanut BAT	Predictive	BAT (Flow-CAST)	%	Positive
	Ji et al. [39]	2023	United Kingdom	61*	ND	5 months-15 years (min-max)	NA	Allergy (OFC +)	tIgE	Predictive	ImmunoCAP (ThermoFisher)	kUA/L	Inverse
									Ara h 2 sIgE	Predictive	ImmunoCAP (ThermoFisher)	kUA/L	Positive
									Ara h 2 sIgE/tIgE	Predictive	ImmunoCAP (ThermoFisher)	%	Positive
									Ara h 6 sIgE	Predictive	ImmunoCAP (ThermoFisher)	kUA/L	Positive
									Ara h 9 sIgE	Predictive	ImmunoCAP (ThermoFisher)	kUA/L	Inverse
	Ruinemans-Koerts et al. [40]	2022	Netherlands	74	5.3 years (median)	3.6–8.6 (IQR)	NA	Allergy (OFC +)	Peanut sIgE	Predictive	ImmunoCAP (ThermoFisher)	kUA/L	Positive
									Ara h 2 sIgE	Predictive	ImmunoCAP (ThermoFisher)	kUA/L	Positive
									Ara h 6 sIgE	Predictive	ImmunoCAP (ThermoFisher)	kUA/L	Positive
									Peanut BAT	Predictive	indirect BAT (Flow2-CAST)	AUC	Positive
									Ara h 2 BAT	Predictive	indirect BAT (Flow2-CAST)	AUC	Positive
									Ara h 6 BAT	Predictive	indirect BAT (Flow2-CAST)	AUC	Positive
	Percival et al. [57]	2020	Australia	36	10.2 years (median)	5.1–17.1 (min-max)	NA	Allergy (OFC +)	Peanut SPT	Predictive	SPT (Stallergenes extract)	mm	Positive
									Peanut sIgE	Predictive	ImmunoCAP (ThermoFisher)	kUA/L	Positive
									Ara h 2 sIgE	Predictive	ImmunoCAP (ThermoFisher)	kUA/L	Positive
									FeNO	Predictive	FeNO (NIOX VERO-Circassia AB)	ppb	Positive
	Kansen et al. [41]	2021	Netherlands	154	27 years (median)	22–38 (IQR)	NA	Allergy (OFC +)	Peanut sIgE	Predictive	ImmunoCAP (ThermoFisher)	kUA/L	Positive
									Ara h 1 sIgE	Predictive	ImmunoCAP (ThermoFisher)	kUA/L	Positive
									Ara h 2 sIgE	Predictive	ImmunoCAP (ThermoFisher)	kUA/L	Positive
									Ara h 3 s IgE	Predictive	ImmunoCAP (ThermoFisher)	kUA/L	Positive
									Ara h 6 sIgE	Predictive	ImmunoCAP (ThermoFisher)	kUA/L	Positive
	Carrette et al. [42]	2023	France	91	6.8 years (median)	5–11.3.3 (IQR)	NA	Allergy (OFC +)	Ara h 2 sIgE	Predictive	ImmunoCAP (ThermoFisher)	kUA/L	Positive
									Peanut BAT	Predictive	BAT (Flow-CAST)	%	Positive
									Peanut sIgE	Predictive	ImmunoCAP (ThermoFisher)	kUA/L	Positive
	Kidon et al. [49]	2021	Israel	110	3.3 years (mean)	1–6 (min-max)	NA	Allergy (OFC +)	Peanut SPT	Predictive	SPT (ALK extract); LPP-MH (Volvani)	mm	Possitive
									Peanut sIgE	Predictive	ImmunoCAP (ThermoFisher)	kUA/L	Possitive
									Ara h 2 sIgE	Predictive	ImmunoCAP (ThermoFisher)	kUA/L	Possitive
	Chua et al. [50]	2021	China	31	4.54 years (median)	3.65–6.46 (95% percentile)	NA	Allergy (OFC +)	Peanut SPT	Predictive	SPT (ALK extract)	mm	Positive
									Ara h 2 sIgE	Predictive	ImmunoCAP (ThermoFisher)	kUA/L	Positive
	Klueber et al. [43]	2023	Luxembourg	26	7.6 years (mean)	3–13 (min-max)	NA	Allergy (OFC +)	Naive CD4 + T cells	Predictive	BAT (Flow-CAST)	%	Inverse
	Ojaniemi et al. [44]	2022	Finland	107	7.18 years (median)	1.17–17.74 (min-max)	NA	Allergy (OFC +)	Ara h 2 sIgE	Predictive	ImmunoCAP (ThermoFisher)	kUA/L	Positive
	Mustillo et al. [55]	2022	Canada	42	26 years (median)	14–54 (min-max)	NA	Allergy (OFC +)	Peanut sIgE	Predictive	ImmunoCAP (ThermoFisher)	kUA/L	Positive
									Peanut sIgE/tIgE	Predictive	ImmunoCAP (ThermoFisher)	kUA/L	Positive
									Ara h 2 sIgE	Predictive	ImmunoCAP (ThermoFisher)	kUA/L	Positive
	Kansen et al. [45]	2021	Netherlands	150	7.9 years (median)	5.4 to 13.0 (IQR)	NA	Allergy (OFC +)	Ara h 2 sIgE	Predictive	ImmunoCAP (ThermoFisher)	kUA/L	Positive
**Pistachio**	Cetinkaya et al. [46]	2021	Turkey	82*	51.9 months (median)*	28.51–87.12 (IQR)*	NA	Allergy (OFC +)	Pistachio SPT	Predictive	SPT (Homemade extract)	mm	Positive
									Pistachio sIgE	Predictive	ImmunoCAP (ThermoFisher)	kUA/L	Positive
									Pistachio sIgE/tIgE	Predictive	ImmunoCAP (ThermoFisher)	NA	Positive
**Sesame**	Santos et al. [36]	2021	United Kingdom, Switzerland, Italy	92	5.1 years (median)	3–9 (IQR)	NA	Allergy (OFC +)	Sesame SPT	Predictive	Commercial tahini paste (Meridian Foods)	mm	Positive
									Sesame sIgE	Predictive	ImmunoCAP (ThermoFisher)	kUA/L	Positive
									Sesame BAT	Predictive	BAT (Flow-CAST)	%	Positive
	Machnes-Maayan et al. [48]	2022	Israel	104	3.93 years (mean)	6 months-17 years (min-max)	NA	Allergy (OFC +)	Sesame SPT	Predictive	SPT (ALK) and natural sesame paste (commercial tahini)	mm	Positive
**Walnut**	Duan et al. [54]	2021	Canada, Austria	107*	8.5 years (mean)	4.6–12.3 (IQR)	NA	Allergy (OFC +)	Jug r 1 sIgE	Predictive	ImmunoCAP (Thermo Fisher)	kU/L	Positive
									Jug r 2 sIgE	Predictive	ImmunoCAP (Thermo Fisher)	kU/L	Positive
									Walnut BAT	Predictive	BAT (Flow-CAST)	%	Positive
	Lee et al. [51]	2021	South Korea	41	3.4 years (median)*	1.25–9.25 (IQR)*	NA	Allergy (OFC +)	Jug r 1 sIgE	Predictive	ISAC ImmunoCAP (Thermofisher)	ISU	Positive
**Wheat**	Al Hawi et al. [52]	2021	Japan	184	1.83 years (median)	1–6.75.75 (min-max)	NA	Allergy (OFC +)	Wheat sIgE	Predictive	ImmunoCAP (ThermoFisher); IMMULITE 2000 3 g Allergy system (Siemens Healthcare Diagnostics)	kUA/L; IU/mL	Positive
									ω5 Gliadin sIgE	Predictive	ImmunoCAP (ThermoFisher); IMMULITE 2000 3 g Allergy system (Siemens Healthcare Diagnostics)	kUA/L; IU/mL	Positive
**Almond**	Santos et al. [36]	2021	United Kingdom, Switzerland, Italy	92	5.1 years (median)	3–9 (IQR)	NA	Allergy (OFC +)	Almond BAT	Predictive	BAT (Flow-CAST)	%	Positive
									Almond SPT	Predictive	Commercial extract (Stallergenes)	mm	Positive

^1^*USA *United States of America. ^2^IQR Interquartile range. ^3^*OFC *Oral food challenge. ^4^*BAT *Basophil activation test, *SPT* Skin prick test, *sIgE* Specific IgE, *tIgE* Total IgE. ^5^*AUC *Area under curve, *ISU* ISAC standardized units, *ppb* Parts per billion. *NA* Not applicable, *ND* Not determined

Regarding patients’ age, 17 studies were conducted in young children (74%) [36, 38, 40, 42–54, 56], three in children and teens up to 18 years old (13%) [44, 48, 57], and three in adults (13%) [35, 41, 55]. According to the number of patients included, they ranged from small cohorts (12 subjects) [35], focused on discovering new Bms [35, 43], to big study populations (981 subjects) [37], when studying contrasted Bms.

Apart from sesame [36, 48] and wheat [52], the majority of the studies were conducted in peanut (73.9%) [35–37, 39–45, 49, 50, 53–57], followed by nuts (26.1%) [36, 38, 46, 47, 51, 54]. Specifically, hazelnut (13%) [36, 47, 54], cashew (13%) [36, 38, 46], walnut (8.7%) [51, 54], pistachio (4.3%) [46] and almond (4.3%) [36] were evaluated.

The most frequently identified Bms were sIgE against both total and molecular allergens (82.6%) [36, 38–42, 44–47, 49–57]. Precisely, 12 studies analyzed sIgE against complete allergen extracts (52.2%) [36, 38, 40–42, 46, 47, 49, 52, 55–57]; while 18 articles evaluated sIgE against molecular components (78.3%) [36, 38–42, 44, 45, 47, 49–57], being Ara h 2 the most frequent allergen (60.9%) [36, 39–42, 44, 45, 49, 50, 53–57]. Otherwise, eight studies evaluated SPT (34.8%) [36, 38, 46, 48–50, 56, 57] determinations with commercial (26.1%) [36, 38, 48–50, 57] or homemade extracts (4.3%) [46], and natural foods (8.7%) [36, 48]. Moreover, six studies analyzed BAT (26.1%) [36–38, 40, 42, 54], with complete extract (17.4%) [36–38, 54] and molecular allergens (8.7%) [36, 40]. Finally, a minority of articles evaluated other Bms, such as naive CD4 T cells [43], genetic factors [35], tIgE [39] and FeNO [57].

### Severity

The severity section had the highest number of articles. We included 25 studies about Bms related to the severity of reaction, performed in 4,799 patients and identifying 44 different Bms (Table 3). Half of the studies were based on clinical response after OFC [37, 38, 56–66], rather than on patients’ clinical history. However, the severity concept was determined heterogeneously among these articles, since some papers considered the most severe clinical pattern anaphylaxis diagnosis [57–59, 67–70] or epinephrine use [56], or both [61]; while others considered non-local systemic symptoms [71–73] or even intensity of oral allergy syndrome (OAS) as a grading system for reactions severity [60]. Most of the studies graded severity based on published severity scales: Sampson et al. classification was the most frequently used [74], in four studies [40, 56, 63, 65]; followed by Ring & Messmer classification [75], in two articles [62, 69]; and Muraro et al. classification [76], in two studies [63, 67]. In addition, mild and severe definitions appeared overlapping among studies [10, 61, 67, 77]. OAS patients were excluded in one article [78], while in other studies they represented the mild reaction group [71–73] or was used for severity grading [60]. The control group was also heterogeneous among articles, since mild allergic patients were used as control in some studies [38, 58, 62, 63, 65, 66, 69, 71, 72, 79], while others included sensitized tolerating patients [56, 59, 61, 64, 77].

**Table 3:** Studies regarding susceptibility to present severe reactions (*n* = 25). Italics* indicate the specific study population for that biomarker (Bm) within the total cohort. A positive association is related to an increase in the Bm according to the pathology/condition evaluated, while an inverse association is linked to a decrease in the Bm

	ARTICLE			STUDIED POPULATION						BIOMARKER				
Food	Reference	Year of publication	Country^1^		Sample size	Age	Range^2^	Control group	Pathology/Condition^3^	Name^4^	Type	Diagnostic technique (manufacturer)	Units^5^	Association
**Almond**	Alves et al. [67]	2022	Portugal		82*	13 years (median)	8–19 (min-max)	Yes	Anaphylaxis	Almond sIgE	Susceptibility	ImmunoCAP (ThermoFisher)	kUA/L	Positive
										Pru p 3 sIgE	Susceptibility	ImmunoCAP (ThermoFisher)	kUA/L	Positive
	Virkud et al. [59]	2019	USA		590	10.2 years (mean)	8.1 (SD)	Yes	Anaphylaxis	Almond sIgE	Susceptibility	ImmunoCAP (ThermoFisher)	kUA/L	Positive
										Almond SPT	Susceptibility	SPT (ND extract)	mm	Positive
										Peanut sIgE	Susceptibility	ImmunoCAP (ThermoFisher)	kUA/L	Positive
										Pistachio sIgE	Susceptibility	ImmunoCAP (ThermoFisher)	kUA/L	Positive
										Pecan sIgE	Susceptibility	ImmunoCAP (ThermoFisher)	kUA/L	Positive
										Walnut sIgE	Susceptibility	ImmunoCAP (ThermoFisher)	kUA/L	Positive
**Cashew**	Goldberg et al. [58]	2024	Israel		74*	8.4 years (median)	6.2–11.6 (IQR)	Yes	Anaphylaxis	Cashew SPT	Susceptibility	SPT (Homemade extract)	mm	Positive
										Ana o 3 sIgE	Susceptibility	ImmunoCAP (ThermoFisher)	kUA/L	Positive
	Röntynen et al. [38]	2022	Finland		72	5.5 years (median)	1.4–16.7 (min-max)	Yes	Moderate-severe reaction	Cashew SPT	Susceptibility	SPT (ALK extract)	mm	Positive
										Cashew BAT	Susceptibility	Flow-CAST (Bülhman)	%	Positive
**Hazelnut**	Alves et al. [67]	2022	Portugal		90*	12 years (median)	3–16 (min-max)	Yes	Anaphylaxis	Bet v 1 sIgE	Susceptibility	ImmunoCAP (ThermoFisher)	kUA/L	Inverse
	Valbuena et al. [63]	2021	Spain		22	8.1 years (mean)	2.7 (SD)	Yes	Severe *vs*. moderate/mild reaction	Cor a 11 sIgE	Susceptibility	ALEX (MacroArrayDX)	kUA/L	Positive
										Cor a 14 sIgE	Susceptibility	ImmunoCAP (ThermoFisher)	kUA/L	Positive
										Hazelnut sIgE	Susceptibility	Immunilte (Siemens)	kUA/L	Positive
										Sunflower seed SPT	Susceptibility	SPT (Leti SLU or Roxall SA or ALK SA)	mm	Positive
**Pecan**	Kubota et al. [68]	2022	Japan		21*	8 years (mean)	2.5–15.5 (range)	Yes	Anaphylaxis	Macadamia sIgE	Susceptibility	ImmunoCAP (ThermoFisher)	kUA/L	Positive
	Goldberg et al. [61]	2021	Israel		98*	8 years (median)	6–11 (min-max)	Yes	Lower respiratory symptoms at OFC	Pecan BAT	Susceptibility	BAT (Homemade)	%	Positive
**Peach**	Urbani et al. [80]	2022	Italy		165	37.8 years (mean)	12.4 (SD)	Yes	Severe reactions in 3 years follow-up	Pla a 3 sIgE	Susceptibility	ImmunoCAP (ThermoFisher)	kUA/L	ND
	Kallen et al. [78]	2023	Iceland, United Kingdom, Netherlands, Poland, Czech Republic, Bulgaria, France, Lithuania, Switzerland, Italy, Greece, Spain, Japan		477	32.2 years (mean)	14.7 (SD)	Yes	Severe symptoms	Pru p 7 sIgE	Susceptibility	ImmunoCAP (ThermoFisher)	kUA/L	Positive
										Cup s 7 sIgE	Susceptibility	ImmunoCAP (ThermoFisher)	kUA/L	Positive
	Ando et al. [71]	2020	Japan		27	13 years (median)	7–20 (min-max)	No	Systemic reaction *vs*. local reaction	Pru p 1 sIgE	Susceptibility	ImmunoCAP (ThermoFisher)	kUA/L	Inverse
										Pru p 4 sIgE	Susceptibility	ImmunoCAP (ThermoFisher)	kUA/L	Inverse
										Pru p 7 sIgE	Susceptibility	ImmunoCAP (ThermoFisher)	kUA/L	Positive
										Alder sIgE	Susceptibility	ImmunoCAP (ThermoFisher)	kUA/L	Inverse
	Deng et al. [72]	2019	China		38	26 years (median)	11–58 (min-max)	Yes	Systemic reaction *vs*. OAS	Pru p 3 sIgE	Susceptibility	ImmunoCAP (ThermoFisher)	kUA/L	Positive
	Asaumi et al. [73]	2019	Japan		90	10 years (median)	8–12 (IQR)	Yes	Systemic *vs*. local reaction	Alder sIgE	Susceptibility	ImmunoCAP (ThermoFisher)	kUA/L	Inverse
										Pru p 1 sIgE	Susceptibility	ImmunoCAP (ThermoFisher)	kUA/L	Inverse
										Pru p 4 sIgE	Susceptibility	ImmunoCAP (ThermoFisher)	kUA/L	Inverse
										Bet v 1 sIgE	Susceptibility	ImmunoCAP (ThermoFisher)	kUA/L	Inverse
**Peanut**	Alves et al. [67]	2022	Portugal		121*	7 years (median)	3–15 (IQR)	Yes	Anaphylaxis	Ara h 2 sIgE	Susceptibility	ImmunoCAP (ThermoFisher)	kUA/L	Positive
										Bet v 1 sIgE	Susceptibility	ImmunoCAP (ThermoFisher)	kUA/L	Inverse
	Santos et al. [37]	2020	United Kingdom		981	ND	5–6 (min-max)	Yes	Severe symptoms	Peanut BAT	Susceptibility	BAT (Homemade)	%	Positive
										Ara h 2 sIgE	Susceptibility	ImmunoCAP (ThermoFisher)	kUA/L	Positive
										Peanut SPT	Susceptibility	SPT (ALK extract)	mm	Positive
										Peanut sIgE	Susceptibility	ImmunoCAP (ThermoFisher)	kUA/L	Positive
										Peanut sIgG4/sIgE	Susceptibility	ImmunoCAP (ThermoFisher)	kUA/L	Positive
	Kaur et al. [56]	2021	Australia		222	8 years (median)	5–12 (min-max)	Yes	Severe symptoms	Peanut sIgE	Susceptibility	ImmunoCAP (ThermoFisher)	kUA/L	Positive
										Ara h 1 sIgE	Susceptibility	ImmunoCAP (ThermoFisher)	kUA/L	Positive
										Ara h 2 sIgE	Susceptibility	ImmunoCAP (ThermoFisher)	kUA/L	Positive
										Ara h 6 sIgE	Susceptibility	ImmunoCAP (ThermoFisher)	kUA/L	Positive
	Percival et al. [57]	2020	Australia		21	11.4 years (median)	5.1–16.5 (min-max)	Yes	Anaphylaxis *vs*. non-anaphylactic symptoms at OFC	FeNO	Prognostic	FeNO (NIOX VERO-Circassia AB)	ppb	Inverse
	Datema et al. [77]	2021	Iceland, United Kingdom, Netherlands, Poland, Czech Republic, Bulgaria, France, Lithuania, Switzerland, Italy, Greece, Spain		393	24.8 years (mean)*	13.7 (SD)*	Yes	Severe *vs*. mild/moderate reaction	Peanut SPT	Susceptibility	SPT (ALK extract)	mm	Positive
										Peanut sIgE	Susceptibility	ImmunoCAP (ThermoFisher)	kUA/L	Positive
										Ara h 1 sIgE	Susceptibility	ISAC ImmunoCAP (Thermofisher)	ISU	Positive
										Ara h 2 sIgE	Susceptibility	ISAC ImmunoCAP (Thermofisher)	ISU	Positive
										Ara h 6 sIgE	Susceptibility	ISAC ImmunoCAP (Thermofisher)	ISU	Positive
										Ara h 3 sIgE	Susceptibility	ISAC ImmunoCAP (Thermofisher)	ISU	Positive
										Ara h 8 sIgE	Susceptibility	ISAC ImmunoCAP (Thermofisher)	ISU	Inverse
	Al-Ahmad et al. [79]	2022	Kuwait		52*	23.6 years (mean)	11.8 (SD)	Yes	Systemic *vs*. local reaction	Peanut sIgE	Susceptibility	ImmunoCAP (ThermoFisher)	kUA/L	Positive
										Ara h 1 sIgE	Susceptibility	ImmunoCAP (ThermoFisher)	kUA/L	Positive
										Ara h 2 sIgE	Susceptibility	ImmunoCAP (ThermoFisher)	kUA/L	Positive
	Datema et al. [64]	2019	Denmark		162	6.5 years (mean)	4.4 (SD)	Yes	Severity	Peanut sIgE	Susceptibility	ImmunoCAP (ThermoFisher)	kUA/L	Positive
										Ara h 1 sIgE	Susceptibility	ImmunoCAP (ThermoFisher)	kUA/L	Positive
										Ara h 2 sIgE	Susceptibility	ImmunoCAP (ThermoFisher)	kUA/L	Positive
										Ara h 3 sIgE	Susceptibility	ImmunoCAP (ThermoFisher)	kUA/L	Positive
	Lang et al. [65]	2023	USA		37	ND	ND	Yes	Severity	Alpha tryptase	Susceptibility	PCR	Copy number	Positive
	Petek et al. [69]	2023	Slovenia		94	12 years (median)	3–18 (min-max)	Yes	Anaphylaxis *vs*. non-anaphylactic symptoms	Ara h 2 sIgE	Susceptibility	ImmunoCAP (ThermoFisher)	kUA/L	Positive
	Cottel et al. [66]	2021	France		56	9.5 years (median)	6.9–14 (min-max)	Yes	Severe *vs*. mild-moderate symptoms	tIgE	Susceptibility	ImmunoCAP (ThermoFisher)	kUA/L	Positive
										Peanut sIgE	Susceptibility	ImmunoCAP (ThermoFisher)	kUA/L	Positive
										Ara h 2 sIgE	Susceptibility	ImmunoCAP (ThermoFisher)	kUA/L	Positive
										Positive control BAT (FcεRI)	Susceptibility	Flow-CAST (Bülhman)	%	Positive
										Positive control BAT (fMLP)	Susceptibility	Flow-CAST (Bülhman)	%	Positive
**Pollen-related plant-food**	Li et al. [70]	2020	China		302	29.5 years (mean)	8–84 (min-max)	Yes	Anaphylaxis *vs*. OAS	Mugwort sIgE	Susceptibility	ImmunoCAP (ThermoFisher)	kUA/L	Positive
										Art v 3 sIgE	Susceptibility	ImmunoCAP (ThermoFisher)	kUA/L	Positive
**Soya**	Caballero et al. [60]	2023	Germany		34	38 years (mean)	ND	No	Intensity of oral tingling/itching at OFC	Bet v 1 peptide GL12 (127-139aa) sIgG4	Susceptibility	In silico peptide array	NA	Positive
**Walnut**	Lyons et al. [10]	2021	Iceland, United Kingdom, Netherlands, Poland, Czech Republic, Bulgaria, France, Lithuania, Switzerland, Italy, Greece, Spain		336	30.4 years (mean)	13.9 (SD)	Yes	Severity	Walnut sIgE	Susceptibility	ImmunoCAP (ThermoFisher)	kUA/L	Positive
	Goldberg et al. [61]	2021	Israel		120*	8 years (median)	6–11 (min-max)	Yes	Epinephrin required reaction at OFC	Walnut BAT	Susceptibility	BAT (Homemade)	%	Positive
**Wheat**	Faihs et al. [62]	2023	Germany		22	53.5 years (median)	25–80 (min-max)	Yes	Severe reaction	Gluten sIgE	Susceptibility	ImmunoCAP (ThermoFisher)	mm	Positive
										Gliadin sIgE	Susceptibility	ImmunoCAP (ThermoFisher)	mm	Positive
										Tryptase	Susceptibility	ImmunoCAP (ThermoFisher)	mg/L	Positive

^1^*USA *United States of America. ^2^*IQR *Interquartile range, *SD* Standard deviation. ^3^*OAS* Oral allergy syndrome, *OFC* Oral food challenge. ^4^*BAT *Basophil activation test, *SPT* Skin prick test, *sIgE* Specific IgE, *sIgG4* Specific IgG4. ^5^*ISU *ISAC standardized units, ppb Parts per billion. *NA* Not applicable, *ND* Not determined

The most studied allergic food was peanut (40%) [37, 56, 57, 64–67, 69, 77, 79] and nuts (32%) [10, 38, 58, 59, 61, 63, 67, 68], followed by peach (20%) [71–73, 78, 80] and, less represented, soya (4%) [60] or wheat (4%) [62]. In addition, general pollen related plant-food was also studied in one article [70]. Accordingly, the most endorsed Bms were Ara h 2 sIgE (28%) [37, 56, 64, 66, 67, 69, 79] and peanut sIgE (28%) [37, 56, 59, 64, 66, 77, 79]. However, Pru p 3 sIgE was only related to severity for peach in one study [72] and for almond in another one [67]. Interestingly, the majority of Bms identified for peach severity were related to mild reactions [71, 73]. Moreover, Bms of mild reactions were also identified for hazelnut [67] and peanut [57, 67, 77], related to PR-10 or profilin sensitization.

A large number of studies identified sIgE (76%) [10, 37, 56, 58, 59, 62–64, 66–69, 71, 73, 77–80], mainly to specific allergens (20%) [58, 62, 69, 78, 80] rather than whole extracts (12%) [10, 59, 68], although most of the studies identified both types of sIgE (44%) [37, 56, 63, 64, 66, 67, 70, 71, 73, 77, 79]. Subsequently, the next most frequent Bms were SPT (20%) [37, 58, 59, 63, 77], using commercial (16%) [37, 59, 63, 77] or homemade extracts (4%) [58]; BAT (16%), analyzing the percentage of activated basophils [37, 38, 61] or the response to positive controls [66]; and other Bms (20%), such as sIgG4/sIgE ratio [37], FeNO [57], tryptase levels [62], the number of α-tryptase copies [65] and Bet v 1 peptide GL12 sIgG4 (127-139aa) epitope [60]. Moreover, further than the study focused on pollen related plant FA [70], where mugwort sIgE and Art v 3 sIgE were directly associated with severe reactions, other pollen derived Bms (Pla a 3 sIgE [80] and Alder sIgE [71]) were also associated with peach allergy severity. Interestingly, some Bms were related to severe reactions to different foods from their origin, such as Pru p 3 sIgE [67] or other whole extract tree nuts sIgE [59] for almond.

### Threshold

We included 17 articles showing association between Bms and reactive thresholds, analyzing a total of 2,431 patients and identifying 47 Bms (Table 4). However, the number of subjects per study was heterogeneous depending on the specific food or the type of Bm investigated. Generally, articles on peanut allergy were those with biggest cohorts [37, 81, 82]; while smaller groups of patients were used for investigating less common allergies, such as cofactor-dependent wheat allergy [62], or for studies employing sophisticated technologies [43, 83].

**Table 4:** Studies about different levels of threshold (*n* = 17). Italics* indicate the specific study population for that biomarker (Bm) within the total cohort. A positive association is related to an increase in the Bm according to the pathology/condition evaluated, while an inverse association is linked to a decrease in the Bm

	ARTICLE		STUDIED POPULATION						BIOMARKER				
Food	Reference	Year of publication	Country^1^	Sample size	Age	Range^2^	Control group	Pathology/Condition^3^	Name^4^	Type	Diagnostic technique (manufacturer)	Units^5^	Association
**Cashew**	Goldberg et al. [58]	2024	Israel	82*	8.4 years (median)	6.2–11.6 (IQR)	No	Threshold during OFC (low to high dose)	Cashew BAT	Predictive	BAT (Flow-CAST)	%	Inverse
									Cashew SPT	Predictive	SPT (Homemade extract)	mm	Inverse
									Ana o 3 sIgE	Predictive	ImmunoCAP (ThermoFisher)	kUA/L	Inverse
	Röntynen et al. [38]	2022	Finland	72	5.5 years (median)	1.4–16.7 (range)	Yes	Threshold during OFC (low to high dose)	Ana o 3 sIgE	Predictive	ImmunoCAP (ThermoFisher)	kUA/L	Inverse
									Cashew BAT	Predictive	BAT (Flow-CAST)	%	Inverse
									Cashew SPT	Predictive	SPT (ALK extract)	mm	Inverse
									Cashew sIgE	Predictive	ImmunoCAP (ThermoFisher)	kUA/L	Inverse
**Peanut**	Santos et al. [37]	2020	United Kingdom	981	ND	5–6 (min-max)	No	Threshold during OFC (low to high dose)	Peanut SPT	Predictive	SPT (ALK extract)	mm	Inverse
									Ara h 2 sIgE	Predictive	ImmunoCAP (ThermoFisher)	kUA/L	Inverse
									Peanut sIgE	Predictive	ImmunoCAP (ThermoFisher)	kUA/L	Inverse
									Peanut IgG4/IgE	Predictive	ImmunoCAP (ThermoFisher)	kUA/L	Inverse
									Peanut BAT	Predictive	BAT (Homemade)	%	Inverse
	Kaur et al. [56]	2021	Australia	89*	8 years (median)	5–12 (IQR)	Yes	Threshold during OFC (low to high dose)	Ara h 1 sIgE	Predictive	ImmunoCAP (ThermoFisher)	kUA/L	Inverse
									Ara h 2 sIgE	Predictive	ImmunoCAP (ThermoFisher)	kUA/L	Inverse
									Ara h 3 sIgE	Predictive	ImmunoCAP (ThermoFisher)	kUA/L	Inverse
									Ara h 6 sIgE	Predictive	ImmunoCAP (ThermoFisher)	kUA/L	Inverse
									Peanut sIgE	Predictive	ImmunoCAP (ThermoFisher)	kUA/L	Inverse
	Ruinemans-Koerts et al. [40]	2021	Netherlands	38*	5.3 years (median)	3.6–8.00 (IQR)	Yes	Threshold during OFC (low to high dose)	Ara h 2 Indirect BAT (EC50)	Predictive	BAT (Flow-CAST)	ng/mL	Positive
	Klueber et al. [43]	2022	Luxemburg	11*	7.5 years (mean)	3–12 (min-max)	Yes	Threshold during OFC (low to high dose)	Intermediate monocyte cells	Predictive	Maxpar Human Immune Monitoring Panel kit (Fluidigm)	N. of events	Inverse
									Myeloid dendritic cells	Predictive	Maxpar Human Immune Monitoring Panel kit (Fluidigm)	N. of events	Inverse
	Elegbede et al. [81]	2018	France, Belgium, Luxemburg	238	8 years (median)	6–11 (IQR)	No	Threshold during OFC (low to high dose)	Ara h 2 sIgE	Predictive	ImmunoCAP (ThermoFisher)	kUA/L	Inverse
									Peanut SPT	Predictive	ND	mm	Inverse
	Berin et al. [83]	2022	USA	74	9.5 years (mean)	4–20 (min-max)	No	Threshold during OFC (low to high dose)	IL4 + CD154 + cells	Predictive	Cytometry (FlowJo Software)	N. of events	Inverse
									Type 2 CD4 + cells	Predictive	Cytometry (FlowJo Software)	N. of events	Inverse
	Suprun et al. [82]	2022	United Kingdom, Australia	331	ND	4–25 (min-max)	Yes	Low, moderate and high cumulative tolerated dose	Peptides of Ara h 1 sIgE (*n* = 33) #	Predictive	BBEA	Signal level	Positive
									Peptides of Ara h 2 sIgE (*n* = 14) #	Predictive	BBEA	Signal level	Positive
									Peptides of Ara h 3 sIgE (*n* = 14) #	Predictive	BBEA	Signal level	Positive
	Dreskin et al. [84]	2019	USA	46*	ND	0.75–3.75 (min-max)	No	Threshold during OFC (low to high dose)	Peanut sIgE	Predictive	ImmunoCAP (ThermoFisher)	kUA/L	Inverse
									Ara h 2 sIgE	Predictive	ImmunoCAP (ThermoFisher)	kUA/L	Inverse
									Linear epitope 5 of Ara h 2 sIgE	Predictive	iPepStar™ peptide microarray platform (JPT Peptide Technologies)	Signal level	Inverse
									Linear epitope 6 of Ara h 2 sIgE	Predictive	iPepStar™ peptide microarray platform (JPT Peptide Technologies)	Signal level	Inverse
	Ruiter et al. [86]	2019	USA	62	17 years (median)	10–24 (IQR)	Yes	Low and high reactive dose	Peanut sIgE	Predictive	ImmunoCAP (ThermoFisher)	kUA/L	Inverse
									Peanut sIgE/tIgE	Predictive	ImmunoCAP (ThermoFisher)	kUA/L	Inverse
									Ara h 2 sIgE	Predictive	ImmunoCAP (ThermoFisher)	kUA/L	Inverse
									Peanut sIgE/IgG4	Predictive	ImmunoCAP (ThermoFisher)	kUA/L	Inverse
									Peanut SPT	Predictive	ND	mm	Inverse
									Peanut specific T cells	Predictive	Cytometry (FlowJo Software)	N. of events	Positive
	Cottel et al. [66]	2020	France	56	12.4 years (median)	7.7–14.1 (IQR)	No	Low and high reactive dose	Ara h 2 sIgE	Predictive	ImmunoCAP (ThermoFisher)	kUA/L	Inverse
									Peanut BAT (10ng/ml)	Predictive	BAT (Flow-CAST)	%	Inverse
									Peanut sIgE	Predictive	ImmunoCAP (ThermoFisher)	kUA/L	Inverse
									Peanut BAT (1ng/ml)	Predictive	BAT (Flow-CAST)	%	Inverse
									Peanut BAT (EC50)	Predictive	BAT (Flow-CAST)	ng/mL	Positive
									Peanut BAT (AUC)	Predictive	BAT (Flow-CAST)	AUC	Inverse
									Peanut BAT (100ng/ml)	Predictive	BAT (Flow-CAST)	%	Inverse
									Normalized (FcεRI) Peanut BAT (10ng/ml)	Predictive	BAT (Flow-CAST)	%	Inverse
									Normalized (FcεRI) Peanut BAT (1ng/ml)	Predictive	BAT (Flow-CAST)	%	Inverse
									Normalized (FcεRI) Peanut BAT (EC50)	Predictive	BAT (Flow-CAST)	ng/mL	Positive
									Normalized (FcεRI) Peanut BAT (AUC)	Predictive	BAT (Flow-CAST)	AUC	Inverse
	Zhang et al. [87]	2022	USA	59	ND	4–14 (min-max)	Yes	Low and high reactive dose	α-diversity of oral microbiota	Predictive	16 S rRNA sequencing	Shannon index	Inverse
									Oral *Veillonella nakazawae* (ASV 1979)	Predictive	16 S rRNA sequencing	ASVs	Positive
									Stool *Bacteroides thetaiotaomicron* (ASV 6829)	Predictive	16 S rRNA sequencing	ASVs	Inverse
**Pistachio**	Goldberg et al. [58]	2024	Israel	74*	8.4 years (median)	6.2–11.6 (IQR)	No	Threshold during OFC (low to high dose)	Pistachio SPT	Predictive	SPT (Homemade extract)	mm	Inverse
									Ana o 3 sIgE	Predictive	ImmunoCAP (ThermoFisher)	kUA/L	Inverse
**Soya**	Caballero et al. [60]	2023	Germany	34	38 years (mean)	ND	No	Threshold during OFC (low to high dose)	Bet v 1 peptide GL12 (127-139aa) sIgG4	Predictive	In silico peptide array	NA	Positive
**Walnut**	Goldberg et al. [61]	2020	Israel	120*	8 years (median)	6–11 (min-max)	No	Threshold during OFC (low to high dose)	Pecan SPT	Predictive	SPT (Homemade extract)	mm	Inverse
									Pecan BAT	Predictive	BAT (Homemade)	%	Inverse
									Walnut BAT	Predictive	BAT (Homemade)	%	Inverse
**Wheat**	Faihs et al. [62]	2023	Germany	22	53.5 years (median)	25–80 (min-max)	No	Threshold during OFC (low to high dose)	Gluten sIgE	Predictive	ImmunoCAP (ThermoFisher)	kUA/L	Inverse
									Gliadin sIgE	Predictive	ImmunoCAP (ThermoFisher)	kUA/L	Inverse
									ω5 Gliadin sIgE	Predictive	ImmunoCAP (ThermoFisher)	kUA/L	Inverse
	Itonaga et al. [85]	2024	Japan	124	2.4 years (median)	1.6–3.8 (IQR)	Yes	Tolerance to low dose	tIgE	Predictive	ImmunoCAP (ThermoFisher)	kUA/L	Inverse
									Wheat sIgE	Predictive	ImmunoCAP (ThermoFisher)	kUA/L	Inverse
									ω5 Gliadin sIgE	Predictive	ImmunoCAP (ThermoFisher)	kUA/L	Inverse

^1^*USA *United States of America. ^2^*IQR *Interquartile range. ^3^*OFC *Oral food challenge. ^4^*AUC *Area under curve, *BAT* Basophil activation test, *EC50* Half maximal effective concentration, *SPT* Skin prick test, *sIgE* Specific IgE, *sIgG4* Specific IgG4. # Specific peptides of Ara h 1 (sIgE): 8, 21, 22, 25, 29, 30, 33, 35, 40, 41, 44, 45, 47, 50, 56, 58, 90, 97, 103, 130, 131, 137, 167, 170, 173, 176, 179, 180, 184, 186, 187, 194, 203; Specific peptides of Ara h 2 (sIgE): 5, 8, 10, 17–19, 21, 30, 31, 36–38, 40, 45; Specific peptides of Ara h 3 (sIgE): 18, 30, 31, 37, 60, 68, 79, 80, 92, 93, 100, 102, 152, 162. ^5^*ASVs *Amplicon variant sequence, *AUC *Area under curve. *NA* Not applicable, *ND* Not determined

Threshold was assessed by analyzing the reactive dose at OFC based on different protocols among studies, even on the same plant food. To classify patients’ threshold some articles used the specific allergen amount [38, 56, 60, 61, 84], continuous numeric transformed variables [58, 81] or ordinary scales, employing the interaction between dose and cofactors [62]; whereas other studies used categorical classifications such as low-high threshold [37, 40, 43, 66, 83, 85–87] or low-moderate-high dose reactivity thresholds [81, 82].

Peanut was the most frequent cause of FA in this section (64.7%) [37, 40, 43, 56, 66, 81–84, 86, 87]; followed by nuts (17.6%) [38, 58, 61], although only one article evaluated more than one nut [58]; wheat (11.8%) [62, 85], where one study considered cofactor-dependent allergy [62]; and soybean (5.9%) [60].

The most frequent Bms were sIgE against molecular allergens (58.8%) [37, 38, 56, 58, 62, 66, 81, 84–86] and to the whole food extract (47.1%) [37, 38, 56, 62, 66, 84–86]. Furthermore, epitope-based array studies identified different peptide sIgE (11.8%) [82, 84] or sIgG4 (5.9%) [60], requiring further validation. Moreover, SPT was evaluated in six articles (35.3%) [37, 38, 58, 61, 81, 86], using commercial [37, 38] and homemade extracts [58, 61], although two studies did not specify the origin [81, 86]. Likewise, BAT was also measured in six articles (35.3%) [37, 38, 40, 58, 61, 66], even using different parameters such as EC50 or area under the curve (AUC) [40, 66]. In addition, novel threshold Bms have been described related to stool and oral microbiome [87] or to specific peripheral blood cell populations [43, 83, 86].

### Follow-up Treatment

We included 15 papers about Bms related to treatment response, performed in 1,687 patients and identifying 53 Bms (Table 5). Among them, 11 studies were focused on OIT (73.3%) [84, 85, 88–96], one using Omalizumab as adjunct therapy [94], whereas four articles evaluated EPIT (26.7%) [83, 97–99]. Treatment response was determined heterogeneously among these articles. Certain studies considered efficacy when demonstrating sustained unresponsiveness [84, 90, 91, 93, 96], others when changing the immunological parameters after desensitization [83, 84, 88, 89, 92–95, 97], and others when increasing the threshold tolerated dose [85, 91, 98, 99]. These differences did not make comparisons difficult, as all the studies had similar endpoints: identifying Bms related to successful treatment response. In five articles there was no control group (33%) [88, 91, 93–95] defined mainly as subjects receiving a placebo.

**Table 5:** Studies about the response to treatments (*n* = 15). Italics* indicate the specific study population for that biomarker (Bm) within the total cohort. A positive association is related to an increase in the Bm according to the pathology/condition evaluated, while an inverse association is linked to a decrease in the Bm

	ARTICLE		STUDIED POPULATION						BIOMARKER				
Food	Reference	Year of publication	Country^1^	Sample size	Age	Range^2^	Control group	Pathology/Condition^3^	Name^4^	Type	Diagnostic technique (manufacturer)	Units^5^	Association
**Hazelnut**	Moraly et al. [88]	2020	France	100	5 years (median)	3–9 (IQR)	No	OIT	Hazelnut sIgE	Treatment response prediction	ImmunoCAP (ThermoFisher)	kUA/L	Inverse
									Cor a 14 sIgE	Treatment response prediction	ImmunoCAP (ThermoFisher)	kUA/L	Inverse
**Peanut**	Jones et al. [89]	2022	USA	146	39.3 months (median)	30.8–44.7 (IQR)	Yes	OIT	Peanut sIgE	Treatment response prediction	ImmunoCAP (ThermoFisher)	kUA/L	Inverse
									Peanut sIgE/tIgE	Treatment response prediction	ImmunoCAP (ThermoFisher)	NA	Inverse
									Ara h 1 sIgE	Treatment response prediction	ImmunoCAP (ThermoFisher)	kUA/L	Inverse
									Ara h 2 sIgE	Treatment response prediction	ImmunoCAP (ThermoFisher)	kUA/L	Inverse
									Ara h 3 sIgE	Treatment response prediction	ImmunoCAP (ThermoFisher)	kUA/L	Inverse
									Ara h 6 sIgE	Treatment response prediction	ImmunoCAP (ThermoFisher)	kUA/L	Inverse
									Peanut BAT	Treatment response prediction	BAT	%	Inverse
									Peanut sIgG4	Treatment response prediction	ImmunoCAP (ThermoFisher)	kUA/L	Positive
									Ara h 1 sIgG4	Treatment response prediction	ImmunoCAP (ThermoFisher)	kUA/L	Positive
									Ara h 2 sIgG4	Treatment response prediction	ImmunoCAP (ThermoFisher)	kUA/L	Positive
									Ara h 3 sIgG4	Treatment response prediction	ImmunoCAP (ThermoFisher)	kUA/L	Positive
									Ara h 6 sIgG4	Treatment response prediction	ImmunoCAP (ThermoFisher)	kUA/L	Positive
	Berin et al. [83]	2021	USA	74	9.5 years (mean)	4–20 (min-max)	Yes	EPIT	IL-4	Treatment response prediction	T cell assays (BD Biosystems)	MFI	Inverse
									IL-10	Treatment response prediction	T cell assays (BD Biosystems)	MFI	Positive
									IL-13	Treatment response prediction	T cell assays (BD Biosystems)	MFI	Inverse
									CCR6+ cells(antigen-specific T cell)	Treatment response prediction	PBMCs assay (BD Biosystems)	%	Positive
	Tsai et al. [90]	2020	USA	120	11 years (median)	1–3 (IQR)	Yes	OIT	Peanut sIgE	Treatment response prediction	ImmunoCAP (ThermoFisher)	kUA/L	Inverse
									Ara h 1 sIgE	Treatment response prediction	ImmunoCAP (ThermoFisher)	kUA/L	Inverse
									Ara h 2 s IgE	Treatment response prediction	ImmunoCAP (ThermoFisher)	kUA/L	Inverse
									Ara h 3 sIgE	Treatment response prediction	ImmunoCAP (ThermoFisher)	kUA/L	Inverse
									Peanut sIgE/tIgE	Treatment response prediction	ImmunoCAP (ThermoFisher)	kUA/L	Inverse
									Peanut sIgG4	Treatment response prediction	ImmunoCAP (ThermoFisher)	kUA/L	Positive
									Peanut sIgG4/sIgE	Treatment response prediction	ImmunoCAP (ThermoFisher)	NA	Positive
									Peanut BAT	Treatment response prediction	BAT	%	Inverse
									Peanut BAT	Sustained unresponsiveness prediction	BAT	%	Inverse
	Scurlock et al. [97]	2021	USA	74	6.2 years (median)	5.2–9.1 (IQR)	Yes	EPIT	Peanut sIgG4	Treatment response prediction	ImmunoCAP (ThermoFisher)	kUA/L	Positive
									Ara h 2 sIgG4	Treatment response prediction	ImmunoCAP (ThermoFisher)	kUA/L	Positive
									Peanut sIgE	Treatment response prediction	ImmunoCAP (ThermoFisher)	kUA/L	Inverse
	Scurlock et al. [84]	2019	USA	47	NA	9–36 months (min-max)	Yes	OIT	Peanut sIgE	Sustained unresponsiveness prediction	ImmunoCAP (ThermoFisher)	kUA/L	Inverse
									Ara h 2 sIgE	Sustained unresponsiveness prediction	ImmunoCAP (ThermoFisher)	kUA/L	Inverse
									Ara h 2 epitope 1 sIgE	Sustained unresponsiveness prediction	Peptides and microarray printing (Intuitive Biosciences)	kUA/L	Inverse
	Bastin et al. [98]	2023	Australia, Canada, Germany, Ireland, USA	301	7 years (median)*	6–9 (IQR)*	Yes	EPIT	Ara h 1 sIgE	Treatment response prediction	ImmunoCAP (ThermoFisher)	kUA/L	Inverse
									Ara h 2 sIgE	Treatment response prediction	ImmunoCAP (ThermoFisher)	kUA/L	Inverse
									Peanut sIgG4/sIgE	Treatment response prediction	ImmunoCAP (ThermoFisher)	NA	Positive
	O’B Hourihane et al. [92]	2020	Ireland, France, Germany, Italy, Spain, Sweden, United Kingdom	175	9.1 years (mean)	3.7 (SD)	Yes	OIT	Peanut sIgG4	Treatment response prediction	ImmunoCAP (ThermoFisher)	kUA/L	Positive
									Peanut sIgE/sIgG4	Treatment response prediction	ImmunoCAP (ThermoFisher)	NA	Inverse
	Davis et al. [93]	2022	USA	12	8.7 years (mean)	5.2–12.2 (min-max)	No	OIT	IL-4	Treatment response prediction	Cell bead array technology (BD Biosciences)	pg/mL	Inverse
									IL-5	Treatment response prediction	Cell bead array technology (BD Biosciences)	pg/mL	Inverse
									IL-9	Treatment response prediction	Cell bead array technology (BD Biosciences)	pg/mL	Inverse
									IL-13	Treatment response prediction	Cell bead array technology (BD Biosciences)	pg/mL	Inverse
									IL-4	Sustained unresponsiveness prediction	Cell bead array technology (BD Biosciences)	pg/mL	Positive
									IL-5	Sustained unresponsiveness prediction	Cell bead array technology (BD Biosciences)	pg/mL	Positive
									IL-9	Sustained unresponsiveness prediction	Cell bead array technology (BD Biosciences)	pg/mL	Inverse
									IL-10	Sustained unresponsiveness prediction	Cell bead array technology (BD Biosciences)	pg/mL	Positive
									IL-13	Sustained unresponsiveness prediction	Cell bead array technology (BD Biosciences)	pg/mL	Inverse
									IL-17	Sustained unresponsiveness prediction	Cell bead array technology (BD Biosciences)	pg/mL	Inverse
	Fleischer et al. [99]	2020	USA, Canada, Germany, Ireland, Australia, France	356	7 years (median)*	6–9 (IQR)*	Yes	EPIT	Peanut sIgE	Treatment response prediction	ImmunoCAP (ThermoFisher)	kUA/L	Inverse
	Yee et al. [94]	2019	USA	13	10 years (median)	8–16 (min-max)	No	Omalizumab + OIT	tIgE	Treatment response prediction	ImmunoCAP (ThermoFisher)	kUA/L	Inverse
									Peanut sIgE	Treatment response prediction	ImmunoCAP (ThermoFisher)	kUA/L	Inverse
									Peanut sIgE/tIgE	Treatment response prediction	ImmunoCAP (ThermoFisher)	NA	Inverse
									Peanut sIgG4	Treatment response prediction	ImmunoCAP (ThermoFisher)	kUA/L	Positive
									Peanut sIgE/sIgG4	Treatment response prediction	ImmunoCAP (ThermoFisher)	NA	Inverse
									Ara h 1 sIgE	Treatment response prediction	ImmunoCAP (ThermoFisher)	kUA/L	Inverse
									Ara h 2 sIgE	Treatment response prediction	ImmunoCAP (ThermoFisher)	kUA/L	Inverse
									Ara h 1 sIgG4	Treatment response prediction	ImmunoCAP (ThermoFisher)	kUA/L	Positive
									Ara h 2 sIgG4	Treatment response prediction	ImmunoCAP (ThermoFisher)	kUA/L	Positive
									Ara h 1 sIgE/sIgG4	Treatment response prediction	ImmunoCAP (ThermoFisher)	NA	Inverse
									Ara h 2 sIgE/sIgG4	Treatment response prediction	ImmunoCAP (ThermoFisher)	NA	Inverse
	Rambo et al. [95]	2023	USA	20	16.2 years (median)	8–17 (min-max)	No	OIT	Ara h 2 sIgE	Treatment response prediction	ISAC ImmunoCAP (ThermoFisher)	kUA/L	Inverse
									Ara h 3 sIgE	Treatment response prediction	ISAC ImmunoCAP (ThermoFisher)	kUA/L	Inverse
									Ara h 6 sIgE	Treatment response prediction	ISAC ImmunoCAP (ThermoFisher)	kUA/L	Inverse
									Peptide 116 of Ara h 1 sIgE/sIgG4	Treatment response prediction	Peptides and microarray printing (JPT Peptide Technology)	NA	Positive
									Peptides of Ara h 3 sIgE/sIgG4 (*n* = 5) #	Treatment response prediction	Peptides and microarray printing (JPT Peptide Technology)	NA	Positive
									Peptide 16 of Ara h 8 sIgE/sIgG4	Treatment response prediction	Peptides and microarray printing (JPT Peptide Technology)	NA	Positive
									Peptide 13 of Ara h 9 sIgE/sIgG4	Treatment response prediction	Peptides and microarray printing (JPT Peptide Technology)	NA	Positive
									Peptide 12 of Ara h 10 sIgE/sIgG4	Treatment response prediction	Peptides and microarray printing (JPT Peptide Technology)	NA	Positive
									Peptides of Ara h 11 sIgE/sIgG4 (*n* = 5) #	Treatment response prediction	Peptides and microarray printing (JPT Peptide Technology)	NA	Positive
									Peptides of Ara h 1 sIgE/sIgG4 (*n* = 15) #	Treatment response prediction	Peptides and microarray printing (JPT Peptide Technology)	NA	Inverse
									Peptides of Ara h 2 sIgE/sIgG4 (*n* = 8) #	Treatment response prediction	Peptides and microarray printing (JPT Peptide Technology)	NA	Inverse
									Peptides of Ara h 3 sIgE/sIgG4 (*n* = 8) #	Treatment response prediction	Peptides and microarray printing (JPT Peptide Technology)	NA	Inverse
									Peptide 5 of Ara h 6 sIgE/sIgG4	Treatment response prediction	Peptides and microarray printing (JPT Peptide Technology)	NA	Inverse
									Peptides of Ara h 9 sIgE/sIgG4 (*n* = 4) #	Treatment response prediction	Peptides and microarray printing (JPT Peptide Technology)	NA	Inverse
									Peptide 6 of Ara h 11 sIgE/sIgG4	Treatment response prediction	Peptides and microarray printing (JPT Peptide Technology)	NA	Inverse
									Peptide 17 of Ara h 5 sIgG4	Treatment response prediction	Peptides and microarray printing (JPT Peptide Technology)	MFI	Positive
									Peptide 23 of Ara h 6 sIgG4	Treatment response prediction	Peptides and microarray printing (JPT Peptide Technology)	MFI	Positive
									Peptide 25 of Ara h 6 sIgG4	Treatment response prediction	Peptides and microarray printing (JPT Peptide Technology)	MFI	Positive
									Peptide 19 of Ara h 5 sIgE	Treatment response prediction	Peptides and microarray printing (JPT Peptide Technology)	MFI	Positive
	Suprun et al. [96]	2024	USA	106	10 years (median)*	8.5–13.5 (IQR)*	Yes	OIT	tIgE	Treatment response prediction	ImmunoCAP (ThermoFisher)	kUA/L	Inverse
									Peanut sIgE	Treatment response prediction	ImmunoCAP (ThermoFisher)	kUA/L	Inverse
									Ara h 1 sIgE	Treatment response prediction	ImmunoCAP (ThermoFisher)	kUA/L	Inverse
									Ara h 2 sIgE	Treatment response prediction	ImmunoCAP (ThermoFisher)	kUA/L	Inverse
									Ara h 3 sIgE	Treatment response prediction	ImmunoCAP (ThermoFisher)	kUA/L	Inverse
									Peanut sIgG4	Treatment response prediction	ImmunoCAP (ThermoFisher)	kUA/L	Positive
									Ara h 2 sIgG4	Treatment response prediction	ImmunoCAP (ThermoFisher)	kUA/L	Positive
									Peptides of Ara h 1 sIgE (*n* = 14) $	Treatment response and sustained unresponsiveness prediction	BBEA	MFI	Inverse
									Peptides of Ara h 2 sIgE (*n* = 9) $	Treatment response and sustained unresponsiveness prediction	BBEA	MFI	Inverse
									Peptides of Ara h 3 sIgE (*n* = 13) $	Treatment response and sustained unresponsiveness prediction	BBEA	MFI	Inverse
									Peptides of Ara h 1 sIgG4 (*n* = 34) $	Treatment response and sustained unresponsiveness prediction	BBEA	MFI	Inverse
									Peptides of Ara h 2 sIgG4 (*n* = 14) $	Treatment response and sustained unresponsiveness prediction	BBEA	MFI	Inverse
									Peptides of Ara h 3 sIgG4 (*n* = 8) $	Treatment response and sustained unresponsiveness prediction	BBEA	MFI	Inverse
									Peanut sIgE	Sustained unresponsiveness prediction	ImmunoCAP (ThermoFisher)	kUA/L	Inverse
									Ara h 2 sIgE	Sustained unresponsiveness prediction	ImmunoCAP (ThermoFisher)	kUA/L	Inverse
									Ara h 1 sIgE	Sustained unresponsiveness prediction	ImmunoCAP (ThermoFisher)	kUA/L	Inverse
									Peanut sIgG4	Sustained unresponsiveness prediction	ImmunoCAP (ThermoFisher)	kUA/L	Inverse
									Peptides of Ara h 1 sIgE (*n* = 20) $	Sustained unresponsiveness prediction	BBEA	MFI	Inverse
									Peptides of Ara h 2 sIgE (*n* = 7) $	Sustained unresponsiveness prediction	BBEA	MFI	Inverse
									Peptide 92 of Ara h 3 sIgE	Sustained unresponsiveness prediction	BBEA	MFI	Inverse
**Wheat**	Itonaga et al. [85]	2024	Japan	124	2.4 years (median)*	1.6–3.8 (IQR)*	Yes	OIT	Wheat sIgE	Treatment response prediction	ImmunoCAP (ThermoFisher)	kUA/L	Inverse
									ω5 Gliadin sIgE	Treatment response prediction	ImmunoCAP (ThermoFisher)	kUA/L	Inverse
	Pourvali et al. [91]	2023	Iran	19	7.42 years (mean)	3.6 (SD)	No	OIT	Wheat sIgE	Sustained unresponsiveness prediction	ImmunoCAP (ThermoFisher)	kUA/L	Inverse
									Wheat sIgE/sIgG4	Sustained unresponsiveness prediction	ImmunoCAP (ThermoFisher)	NA	Inverse

^1^*USA *United States of America. ^2^*IQR *Interquartile range, *SD *Standard deviation. ^3^*EPIT *Epicutaneous immunotherapy, *OIT* Oral immunotherapy. ^4^*BAT *Basophil activation test, *sIgE* Specific IgE, *sIgG4* Specific IgG4, *tIgE* Total IgE. # Specific peptides of Ara h 3 (sIgE/sIgG4, positive): 4, 5, 49, 50, 61; Specific peptides of Ara h 11 (sIgE/sIgG4, positive): 1, 5, 16–18; Specific peptides of Ara h 1 (sIgE/sIgG4, inverse): 5–7, 15–22, 28–31; Specific peptides of Ara h 2 (sIgE/sIgG4, inverse): 6–8, 17, 28–31; Specific peptides of Ara h 3 (sIgE/sIgG4, inverse): 1–3, 55, 56, 95–97; Specific peptides of Ara h 9 (sIgE/sIgG4, inverse): 6, 7, 9, 16. $ Specific peptides of Ara h 1 (sIgE): 8, 25, 29, 30, 33, 35, 40, 41, 44, 45, 47, 50, 56, 58, 90, 130, 131, 170, 176, 184, 186, 187, 194, 203; Specific peptides of Ara h 2 (sIgE): 5, 8, 10, 17–19, 21, 36–38, 101; Specific peptides of Ara h 3 (sIgE): 18, 30, 31, 37, 68, 79, 80, 92, 93, 100, 102, 152, 162; Specific peptides of Ara h 1 (sIgG4): 8, 15, 21, 22, 25, 29, 30, 33, 35, 40, 41, 44, 45, 47, 50, 56, 58, 90, 97, 103, 130, 131, 137, 167, 170, 173, 176, 179, 180, 184, 186, 187, 194, 203; Specific peptides of Ara h 2 (sIgG4): 5, 8, 10, 14, 17, 19, 21, 30, 36–38, 40, 43, 45; Specific peptides of Ara h 3 (sIgG4): 18, 30, 31, 60, 80, 92, 93, 162. ^5^*MFI *Median fluorescence intensity. *BBEA* Bead-based epitope assay, *PBMCs* Peripheral blood mononuclear cells, *NA* Not applicable, *ND* Not determined

All studies were performed in pediatric cohorts, although two studies also incorporated young adults [83, 95]. The majority of the studies were carried out in America (56%) [83, 84, 89, 90, 93–97], two were performed in Europe (13.3%) [88, 92], and two in several continents (13.3%) [98, 99].

The most frequent Bms were whole extract sIgE (66,7%) [84, 85, 88–91, 94, 96, 97, 99] and molecular sIgE (60%) [84, 85, 88–90, 94–96, 98]. In addition, six articles identified IgG4 as a Bm (40%) [89, 90, 92, 94, 96, 97]. Precisely, treatment efficacy is related to a sIgE decrease [84, 89, 90, 94–96, 98] and a sIgG4 increase for specific allergens [89, 94, 96, 97] and for whole extract [89, 90, 92, 94, 96, 97]. Even more, the decrease in sIgE/tIgE also indicated good tolerance [89, 90, 94]. On the other hand, some studies identified sophisticated Bms such as T cell assays and peptide microarrays [83, 84, 93, 95], or epitope array [95, 96]. In addition, interleukins [83, 93] and sIgE/IgG4 ratio against specific peptides [95] were also characterized.

### Overall Risk-of-bias and Certainty

All articles included were assessed for risk-of-bias using tools adapted to their specific study design. For non-randomized studies (Table S2), the overall risk-of-bias was predominantly moderate, mainly due to confounding and certain limitations in outcome measurement and participant selection. In contrast, classification of exposures, deviations from intended interventions, selection of results, and handling of missing data were generally considered to be at low risk of bias. On the other hand, for studies of diagnostic accuracy (Table S3), the overall risk ranged from low to moderate, primarily due to non-optimal reference standards and issues in patient selection, as well as unclear flow and timing of procedures. Nonetheless, both clinical applicability and validity of the index test were found to be robust. Moreover, for cross-sectional studies (Table S4), the overall risk-of-bias was mostly rated as moderate, primarily due to sample size and justification, followed by issues related to participant selection, risk of non-response bias, and some concerns about measurement validity and reliability. In contrast, domains such as clear aims and justification, appropriate study design, statistical methods and reporting, and ethics and disclosures were consistently rated as low risk. Finally, the follow-up trials (Table S5) showed a low risk-of-bias across all categories. Altogether the body of evidence is characterized by a moderate (*n* = 45) and a low (*n* = 26) risk-of-bias, supporting an intermediate level of confidence in the overall conclusions.

In addition, all the studies were systematically evaluated using the GRADE approach to determine the certainty of evidence on the results (Table S6). The overall quality of evidence varied among low (*n* = 4), moderate (*n* = 59) and high (*n* = 8). The most frequent cause of degradation was imprecision, mainly due to moderate or small sample size. Other relevant factors that influenced the degree of certainty were the risk-of-bias and indirectness, associated with the design of the studies (especially the diagnostic method) and the lack of clinical validation of the results. Inconsistency and publication bias were generally low in all articles. Overall, the scope and robustness of the results were considered moderate, reflecting the exploratory nature of most of the studies.

## Discussion

A total of 71 original articles about Bms in plant FA have been systematically reviewed and summarized. Surprisingly, although the prevalence of plant FA has been increasing globally [100], the number of studies about Bms seems to be limited and has been stable in the last few years. Nevertheless, our vision can be narrowed by the studied time range and the terms used as search criteria.

From a geographical perspective, only a restricted number of countries are researching plant FA Bms, with Europe and North America being the main areas where it has been investigated. No studies from Africa or South America were identified, which may be related to economic constraints or lower prevalences, although these are regions where plant-based foods are commonly consumed. Except for Europe, where studies were more relocated, in North America, Asia or Oceania research comes predominantly from the USA, Japan and Australia, respectively. Moreover, many of these studies were conducted using the same cohort of patients, highlighting the need for new investigations that include multiple countries and diverse geographical regions. On the other hand, most of the reviewed articles were focused on peanuts and nuts, indicating a notable lack of research on fruits, vegetables, and seeds. However, this may partly be a limitation of the search strategy and/or the inclusion and exclusion criteria applied.

Age population is also a relevant factor in plant FA, since most of the Bms studies were settled in pediatric populations. Comparative studies between children and adults are scarce [101–103], despite their potential to provide valuable insights on age-related differences in FA manifestations and mechanisms. Importantly, there are also age-related methodological limitations that may influence the applicability of Bms, as invasive sampling procedures often face greater ethical and practical constraints in pediatric settings, which may restrict the range of parameters assessed and influence study design. Therefore, this review evidences that recently published research on plant FA Bms has been largely conducted in pediatric peanut allergic patients from Europe and North America.

**Fig. 3:**
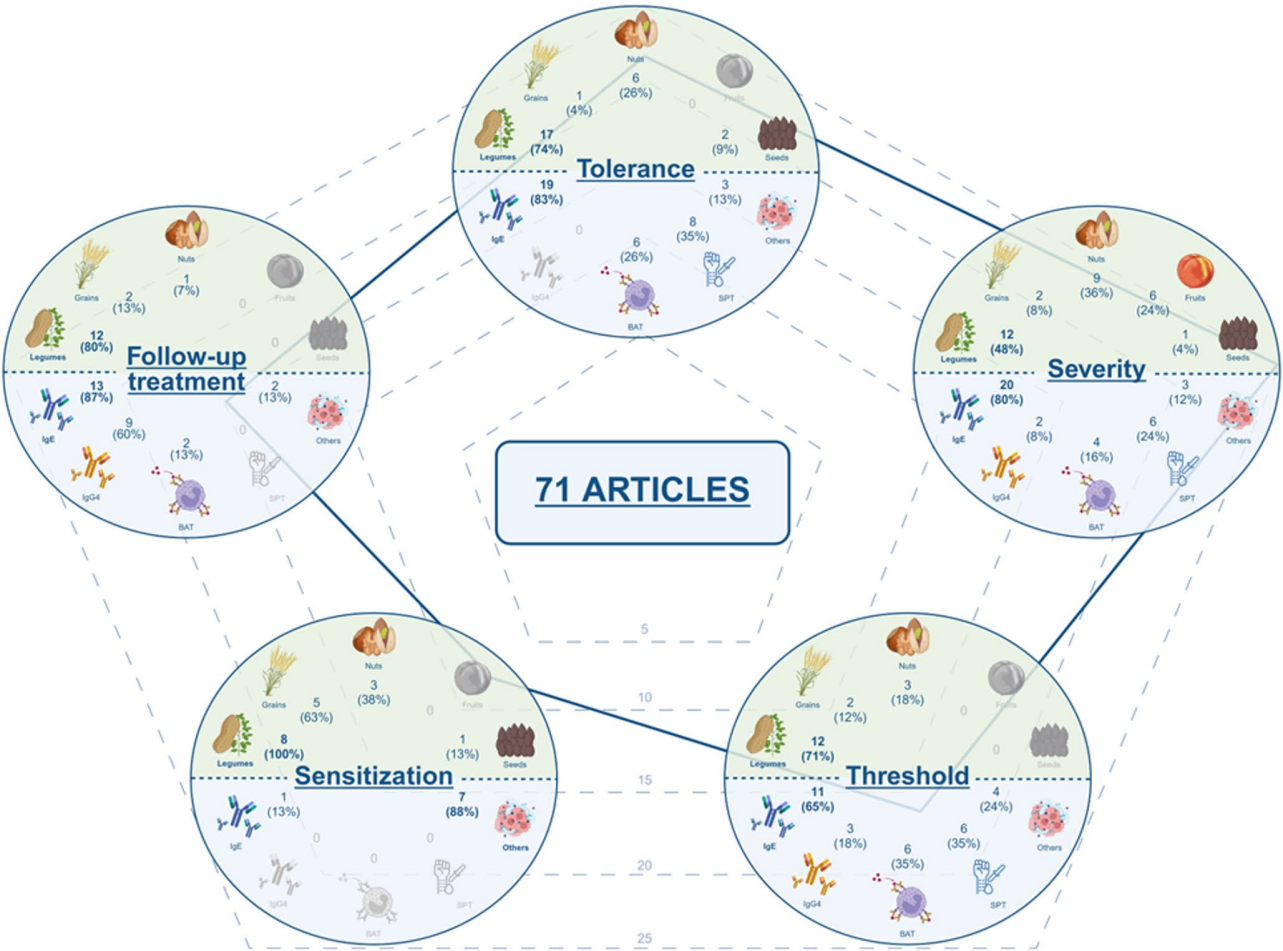
Comparison of allergenic sources and types of biomarkers (Bm) studied across the five thematic sections. Each section includes the number and the percentage of articles describing the Bm/allergenic sources in that section (most of them describe several Bm/allergenic sources). The most frequent Bm/allergenic source is highlighted in bold. The radial graph indicates the number of articles covered in each thematic section. Image created with Biorender.com

To address some of the main clinical questions in plant FA, this review has been structured into five thematic sections (Fig. 3): sensitization acquisition (sensitization), prediction of tolerance (tolerance), susceptibility to develop severe reactions (severity), clinical response to different threshold levels (threshold) or response to validated or non-validated treatments (follow-up treatment). Most of the studies were related to severity and tolerance, and a few to sensitization, suggesting that the classical question of clinical relevance is still a hot topic. However, the low number of articles in the sensitization section could also be influenced by limitations in the search strategy, as it did not include the term “sensitisation”.

Across the five sections, peanut was the most represented food, followed distantly by nuts and grains. There is a notable absence of fruits, only represented by peach in the severity section. In contrast, wheat allergy was represented in all sections, predominantly in sensitization, probably due to its ubiquity more than for its prevalence [104]. In the sensitization section, studies focused on different plant foods were reviewed by repeating a similar list of plant food allergens reported in other sections. No articles on vegetables were found, probably due to limitations in the terms used in our search strategy.

The most frequent type of Bm identified across the different sections was molecular sIgE, closely followed by sIgE against the whole allergenic source. However, the sensitization section was where new Bms (some of them related to atopic dermatitis) were most extensively investigated: such as stratum corneum hydration (SCH), transepithelial water loss (TEWL), plasma and fecal metabolites, and microbiota composition. Although microbiota and FA is a growing interest topic [105, 106], microbiota-related Bms were only identified in one study of sensitization [27] and one study of threshold [87]. The presence of sIgG4, sIgE/sIgG4 ratios or interleukins were more frequent in the follow-up treatment section than in others; whereas cellular response, mainly basophils, was preferentially observed in tolerance, severity and threshold. Precisely, BAT was implicated in different types of Bms, from the percentage of activated basophils incubated with whole extract associated with tolerance [36–38, 42, 47, 48, 51, 88], threshold [37, 38, 61] or severity [61], to the normalized percentage of basophils, the AUC or the EC50, exclusively associated with threshold predictivity [40, 66]. The use of molecular allergens in BAT was mainly identified related to peanut tolerance [36, 40]. On the other hand, apart from the “classical” Bms, recent studies have identified others, such as genes [35], FeNO [57], immune cells [43, 83, 86], interleukins [83, 93], and allergen epitopes [60, 82, 84, 95, 96], although further research is needed.

Overall, studies based on molecular diagnosis identified mainly sIgE and BAT as Bms. Actually, CRD is the cornerstone of precision medicine in FA [107]. The usefulness of CRD is unquestionable, leading to a more accurate diagnosis, stratifying risk or providing more specific management advice [12, 108] and, thus, improving patient safety and quality of life. Certain molecular sIgE determinations are strongly recommended in clinical guidelines as tolerance Bms, such as Ara h 2, Ana o 3 or Cor a 14 sIgE in patients with a history of suspected IgE-mediated allergy to peanut, cashew or hazelnut, respectively [8]. In our review, the predictive role of these Bms has been supported not only in tolerance, but also in FA severity and threshold, although in the latter case only for Ara h 2 and Ana o 3 sIgE, since Cor a 14 sIgE was not identified as a threshold Bm. Precisely, Cor a 14 sIgE was not endorsed by all the studies focused on it. In a Japanese children cohort, Inoue et al. [47] identified high Cor a 9 and low Cor a 1 sIgE levels related to hazelnut allergy, but Cor a 14 sIgE was not significantly associated with hazelnut allergy.

Remarkably, positive results or high levels of the identified Bms were related to the pathology with very rare exceptions [39, 41, 84, 98], such as sensitization, clinical reactivity, more severe reaction or lower threshold. In turn, predominantly lower levels of the identified Bms were related to treatment response, probably due to the outcomes studied. Moreover, Bm identification can be influenced by the selected studied sample. Molecular Bms are a clear example, given that they can be biased by the predominant patient sample serotype. This aspect may potentially compromise the external validation of Bms and contribute to delaying the global access to CRD, although it should not be overlooked that Bms in allergy are more probabilistic than determinant [109]. On the other hand, although CRD is considered a game changer in diagnosis and management of plant FA, “classical” Bms have been identified among the most frequent, such as whole source sIgE, SPT or tIgE. These types of Bms highlight the accessibility of characterizing plant FA in many populations and locations. Even more, in many cases, the Bm identification is determined by the selected studied sample and requires external validation. For example, severity is defined differently in the reviewed articles, and in half of them it is based on symptoms reported by patients.

The assessment of risk-of-bias and certainty indicated an intermediate level of confidence in the overall conclusions of this review. The scope and strength of the evidence were considered moderate, consistent with the exploratory nature of many of the included studies. For these analyses, a hybrid strategy combining AI and human review was implemented. This approach demonstrated clear advantages in terms of efficiency, reproducibility, and reduction of subjective variability compared to fully manual assessments. However, limitations remain, as linguistic models may misinterpret nuanced methodological details or context-specific criteria. Human oversight is therefore essential to ensure accuracy and methodological rigor. While this strategy appears promising for large-scale evidence syntheses and seems encouraging for future reviews, its current role should be considered complementary to expert judgment, not a substitute for it. Future improvements in AI reliability may enhance its utility, but consensus-based oversight remains indispensable at present.

In conclusion, this systematic review highlights the critical role of Bms in advancing precision medicine for plant FA, while revealing significant gaps that limit their global applicability, underscoring that the identified Bms are far from meeting the standards required by the in vitro diagnostic regulation [110], which entails the use of Bms specifically validated technically and clinically for a given intended use when sample-dependent variations are common. Despite the recognized utility of CRD, current testing remains heavily skewed toward peanut allergy and pediatric cohorts in Europe and North America, leaving other plant foods and diverse populations underrepresented. Emerging Bms offer promising avenues for future research but require rigorous validation and standardization before clinical integration. Furthermore, the heterogeneity observed across studies highlights the need for multicenter, and geographically inclusive, research with harmonized methodologies to ensure validity. Bridging these gaps will ultimately be critical to facilitate access to precision diagnostic tools and optimize individualized management strategies, thereby improving the safety and quality of life for patients worldwide.

## Supplementary Information

Below is the link to the electronic supplementary material.

Supplementary Material 1

## Data Availability

No datasets were generated or analysed during the current study.

## References

[1] Arasi S, Mennini M, Valluzzi R et al (2018) Precision medicine in food allergy. Curr Opin Allergy Clin Immunol 18(5):438–443. 10.1097/ACI.0000000000000465 30015641 10.1097/ACI.0000000000000465

[2] Miller RL, Grayson MH, Strothman K (2021) Advances in asthma: new understandings of asthma’s natural history, risk factors, underlying mechanisms, and clinical management. J Allergy Clin Immunol 148:1430–1441. 10.1016/j.jaci.2021.10.001 34655640 10.1016/j.jaci.2021.10.001

[3] Muraro A, Lemanske RF, Castells M et al (2017) Precision medicine in allergic disease-food allergy, drug allergy, and anaphylaxis-PRACTALL document of the European Academy of Allergy and Clinical Immunology and the American Academy of Allergy, Asthma and Immunology. Allergy 72:1006–1021. 10.1111/all.13132 28122115 10.1111/all.13132

[4] Spolidoro GCI, Amera YT, Ali MM et al (2023) Frequency of food allergy in Europe: an updated systematic review and meta-analysis. Allergy 78:351–368. 10.1111/all.15560 36271775 10.1111/all.15560 PMC10099188

[5] Baseggio Conrado A, Patel N, Turner PJ (2021) Global patterns in anaphylaxis due to specific foods: a systematic review. J Allergy Clin Immunol 148:1515-1525.e3. 10.1016/j.jaci.2021.03.048 33940057 10.1016/j.jaci.2021.03.048 PMC8674817

[6] Lyons SA, Clausen M, Knulst AC et al (2020) Prevalence of food sensitization and food allergy in children across Europe. J Allergy Clin Immunol Pract 8:2736-2746.e9. 10.1016/j.jaip.2020.04.020 32330668 10.1016/j.jaip.2020.04.020

[7] Lyons SA, Burney PGJ, Ballmer-Weber BK et al (2019) Food allergy in adults: substantial variation in prevalence and causative foods across Europe. J Allergy Clin Immunol Pract 7:19205–1928.e11. 10.1016/j.jaip.2019.02.044 30898689 10.1016/j.jaip.2019.02.044

[8] Santos AF, Riggioni C, Agache I et al (2023) EAACI guidelines on the diagnosis of IgE-mediated food allergy. Allergy 78:3057–3076. 10.1111/all.15902 37815205 10.1111/all.15902

[9] Vereda A, van Hage M, Ahlstedt S et al (2011) Peanut allergy: clinical and immunologic differences among patients from 3 different geographic regions. J Allergy Clin Immunol 127:603–607. 10.1016/j.jaci.2010.09.010 21093026 10.1016/j.jaci.2010.09.010

[10] Lyons SA, Datema MR, Le T-M et al (2021) Walnut allergy across Europe: distribution of allergen sensitization patterns and prediction of severity. The Journal of Allergy and Clinical Immunology: In Practice 9:225-235.e10. 10.1016/j.jaip.2020.08.051 32916320 10.1016/j.jaip.2020.08.051

[11] Le T-M, Bublin M, Breiteneder H et al (2013) Kiwifruit allergy across Europe: clinical manifestation and IgE recognition patterns to kiwifruit allergens. J Allergy Clin Immunol 131:164–171. 10.1016/j.jaci.2012.09.009 23141741 10.1016/j.jaci.2012.09.009

[12] Dramburg S, Hilger C, Santos AF et al (2023) EAACI molecular allergology user’s guide 2.0. Pediatr Allergy Immunol 34(Suppl 28):e13854. 10.1111/pai.13854 37186333 10.1111/pai.13854

[13] Datema MR, Zuidmeer-Jongejan L, Asero R et al (2015) Hazelnut allergy across Europe dissected molecularly: a EuroPrevall outpatient clinic survey. J Allergy Clin Immunol 136:382–391. 10.1016/j.jaci.2014.12.1949 25772593 10.1016/j.jaci.2014.12.1949

[14] Flores Kim J, McCleary N, Nwaru BI et al (2018) Diagnostic accuracy, risk assessment, and cost-effectiveness of component-resolved diagnostics for food allergy: a systematic review. Allergy 73:1609–1621. 10.1111/all.13399 29319184 10.1111/all.13399 PMC6055682

[15] Bergmann MM, Santos AF (2024) Basophil activation test in the food allergy clinic: its current use and future applications. Expert Rev Clin Immunol 20:1297–1304. 10.1080/1744666X.2024.2336568 38591129 10.1080/1744666X.2024.2336568

[16] Lewis SA, Peters B (2023) T-cell epitope discovery and single-cell technologies to advance food allergy research. J Allergy Clin Immunol 151:15–20. 10.1016/j.jaci.2022.10.025 36411114 10.1016/j.jaci.2022.10.025 PMC9825656

[17] Satitsuksanoa P, Daanje M, Akdis M et al (2021) Biology and dynamics of B cells in the context of IgE-mediated food allergy. Allergy 76:1707–1717. 10.1111/all.14684 33274454 10.1111/all.14684

[18] Gunawardhana K, Raygoza PM, Yang C, Mohamed E (2025) Immunotherapeutic approaches to peanut allergy treatment-pre-clinical and clinical studies: a comprehensive review. J Clin Med 14:1902. 10.3390/jcm14061902 40142710 10.3390/jcm14061902 PMC11943093

[19] Yepes-Nuñez JJ, Zhang Y, Roqué i Figuls M et al (2015) Immunotherapy (oral and sublingual) for food allergy to fruits. Cochrane Database Syst Rev (2):CD010522. 10.1002/14651858.CD010522.pub2 10.1002/14651858.CD010522.pub2 PMC7004415 26558953

[20] Mueller B, Reider N, Demir H et al (2025) Structured fresh apple consumption for birch pollen food allergy syndrome in an uncontrolled phase II/III trial. J Allergy Clin Immunol Pract 13:2777-2786.e3. 10.1016/j.jaip.2025.06.030 40633686 10.1016/j.jaip.2025.06.030

[21] Page MJ, McKenzie JE, Bossuyt PM et al (2021) The PRISMA 2020 statement: an updated guideline for reporting systematic reviews. BMJ 372:n71. 10.1136/bmj.n71 33782057 10.1136/bmj.n71 PMC8005924

[22] FDA-NIH Biomarker Working Group (2016) BEST (Biomarkers, EndpointS, and other Tools) Resource. Food and Drug Administration (US), Silver Spring (MD) 27010052

[23] OpenAI (2023) ChatGPT (March 14 version) [language model]. https://chat.openai.com

[24] Tomlinson E, Cooper C, Davenport C et al (2024) Common challenges and suggestions for risk of bias tool development: a systematic review of methodological studies. J Clin Epidemiol 171:111370. 10.1016/j.jclinepi.2024.111370 38670243 10.1016/j.jclinepi.2024.111370

[25] Downes MJ, Brennan ML, Williams HC, Dean RS (2016) Development of a critical appraisal tool to assess the quality of cross-sectional studies (AXIS). BMJ Open 6:e011458. 10.1136/bmjopen-2016-011458 27932337 10.1136/bmjopen-2016-011458 PMC5168618

[26] Guyatt GH, Oxman AD, Vist GE et al (2008) GRADE: an emerging consensus on rating quality of evidence and strength of recommendations. BMJ 336:924–926. 10.1136/bmj.39489.470347.AD 18436948 10.1136/bmj.39489.470347.AD PMC2335261

[27] Lee-Sarwar KA, Chen Y-C, Lasky-Su J et al (2023) Early-life fecal metabolomics of food allergy. Allergy 78:512–521. 10.1111/all.15602 36448508 10.1111/all.15602 PMC10590492

[28] Goleva E, Calatroni A, LeBeau P et al (2020) Skin tape proteomics identifies pathways associated with transepidermal water loss and allergen polysensitization in atopic dermatitis. J Allergy Clin Immunol 146:1367–1378. 10.1016/j.jaci.2020.04.022 32360271 10.1016/j.jaci.2020.04.022 PMC7606732

[29] Lee-Sarwar K, Kelly RS, Lasky-Su J et al (2019) Dietary and plasma polyunsaturated fatty acids are inversely associated with asthma and atopy in early childhood. The Journal of Allergy and Clinical Immunology: In Practice 7:529-538.e8. 10.1016/j.jaip.2018.07.039 30145365 10.1016/j.jaip.2018.07.039 PMC6400588

[30] Ran Z, Wang B, Zhang S-Y (2024) Associations of exposure to metals with total and allergen-specific IgE: an NHANES analysis (2005–2006). Sci Total Environ 906:167385. 10.1016/j.scitotenv.2023.167385 37777136 10.1016/j.scitotenv.2023.167385

[31] Wärnberg Gerdin S, Lie A, Asarnoj A et al (2022) Impaired skin barrier and allergic sensitization in early infancy. Allergy 77:1464–1476. 10.1111/all.15170 34738238 10.1111/all.15170

[32] Tsilochristou O, du Toit G, Sayre PH et al (2019) Association of *Staphylococcus aureus* colonization with food allergy occurs independently of eczema severity. J Allergy Clin Immunol 144:494–503. 10.1016/j.jaci.2019.04.025 31160034 10.1016/j.jaci.2019.04.025 PMC12351383

[33] Tedner SG, Söderhäll C, Konradsen JR et al (2021) Extract and molecular-based early infant sensitization and associated factors-a PreventADALL study. Allergy 76:2730–2739. 10.1111/all.14805 33751598 10.1111/all.14805

[34] Tran NLH, Ly NTM, Trinh HKT et al (2024) Prediction of food sensitization in children with atopic dermatitis based on disease severity and epidermal layer impairment. Int Arch Allergy Immunol 185:43–55. 10.1159/000533492 37899044 10.1159/000533492

[35] Ehlers AM, den Hartog Jager CF, Knulst AC, Otten HG (2021) Distinction between peanut allergy and tolerance by characterization of B cell receptor repertoires. Allergy 76:2753–2764. 10.1111/all.14897 33969502 10.1111/all.14897 PMC8453529

[36] Santos AF, Bergmann M, Brough HA et al (2021) Basophil activation test reduces oral food challenges to nuts and sesame. The Journal of Allergy and Clinical Immunology: In Practice 9:2016-2027.e6. 10.1016/j.jaip.2020.12.039 33385591 10.1016/j.jaip.2020.12.039 PMC8110244

[37] Santos AF, Du Toit G, O’Rourke C et al (2020) Biomarkers of severity and threshold of allergic reactions during oral peanut challenges. J Allergy Clin Immunol 146:344–355. 10.1016/j.jaci.2020.03.035 32311390 10.1016/j.jaci.2020.03.035 PMC7417812

[38] Röntynen P, Kukkonen K, Savinko T, Mäkelä MJ (2022) Optimizing tools for evaluating challenge outcomes in children with cashew nut allergy. Ann Allergy Asthma Immunol Off Publ Am Coll Allergy Asthma Immunol 128:270–278. 10.1016/j.anai.2021.12.006 10.1016/j.anai.2021.12.006 34896310

[39] Ji C, Huang Y, Yeung LH et al (2023) Ara h 2-specific IgE presence rather than its function is the best predictor of mast cell activation in children. The Journal of Allergy and Clinical Immunology: In Practice 11:1154-1161.e3. 10.1016/j.jaip.2022.12.026 36581066 10.1016/j.jaip.2022.12.026

[40] Ruinemans-Koerts J, Brouwer ML, Schmidt-Hieltjes Y et al (2022) The indirect basophil activation test is a safe, reliable, and accessible tool to diagnose a peanut allergy in children. J Allergy Clin Immunol Pract 10:1305-1311.e3. 10.1016/j.jaip.2021.12.040 35074603 10.1016/j.jaip.2021.12.040

[41] Kansen HM, van Erp FC, Knulst AC et al (2021) Accurate prediction of peanut allergy in one-third of adults using a validated Ara h 2 cutoff. The Journal of Allergy and Clinical Immunology: In Practice 9:1667-1674.e3. 10.1016/j.jaip.2020.11.024 33248282 10.1016/j.jaip.2020.11.024

[42] Carrette M, Couderc L, Bubenheim M et al (2023) The combination of Ara h 2-sIgE and basophil activation test could be an alternative to oral food challenge in cases of suspected peanut allergy. Pediatr Allergy Immunol 34:e14007. 10.1111/pai.14007 37622254 10.1111/pai.14007

[43] Klueber J, Czolk R, Codreanu-Morel F et al (2023) High-dimensional immune profiles correlate with phenotypes of peanut allergy during food-allergic reactions. Allergy 78:1020–1035. 10.1111/all.15408 35700055 10.1111/all.15408

[44] Ojaniemi I, Salmivesi S, Tikkakoski A et al (2022) Are peanut oral food challenges still useful? An evaluation of children with suspected peanut allergy, sensitization to Ara h 2 and controlled asthma. Allergy Asthma Clin Immunol 18:100. 10.1186/s13223-022-00743-6 36451230 10.1186/s13223-022-00743-6 PMC9714138

[45] Kansen HM, van Erp FC, Meijer Y et al (2021) Diagnostic accuracy of Ara h 2 for detecting peanut allergy in children. Clin Exp Allergy 51:1069–1079. 10.1111/cea.13987 34288182 10.1111/cea.13987 PMC8456915

[46] Cetinkaya PG, Karaguzel D, Esenboğa S et al (2021) Pistachio and cashew nut allergy in childhood: predictive factors towards development of a decision tree. Asian Pac J Allergy Immunol 39:53–61. 10.12932/AP-281018-0429 31310145 10.12932/AP-281018-0429

[47] Inoue Y, Sato S, Takahashi K et al (2020) Component-resolved diagnostics can be useful for identifying hazelnut allergy in Japanese children. Allergol Int 69:239–245. 10.1016/j.alit.2019.10.001 31680009 10.1016/j.alit.2019.10.001

[48] Machnes-Maayan D, Yahia SH, Frizinsky S et al (2022) A clinical pathway for the diagnosis of Sesame allergy in children. World Allergy Organ J 15:100713. 10.1016/j.waojou.2022.100713 36440465 10.1016/j.waojou.2022.100713 PMC9685351

[49] Kidon MI, Yahia SH, Machnes-Maayan D et al (2021) Diagnosis of peanut allergy in preschool children: the impact of skin testing with a novel composition of peanuts. Front Pediatr 9:739224. 10.3389/fped.2021.739224 34917557 10.3389/fped.2021.739224 PMC8670606

[50] Chua GT, Chong PC, Au EY et al (2021) Skin Prick testing a better predictor than blood testing for the diagnosis of peanut allergy in Chinese children. Asian Pac J Allergy Immunol 39:241–248. 10.12932/AP-110319-0519 31310149 10.12932/AP-110319-0519

[51] Lee J, Jeong K, Jeon S-A, Lee S (2021) Component resolved diagnosis of walnut allergy in young children: *jug r 1* as a major walnut allergen. Asian Pac J Allergy Immunol 39:190–196. 10.12932/AP-161118-0443 31175720 10.12932/AP-161118-0443

[52] Al Hawi Y, Nagao M, Furuya K et al (2021) Agreement between predictive, allergen-specific IgE values assessed by ImmunoCAP and IMMULITE 2000 3gAllergy™ assay systems for milk and wheat allergies. Allergy Asthma Immunol Res 13:141–153. 10.4168/aair.2021.13.1.141 33191682 10.4168/aair.2021.13.1.141 PMC7680830

[53] Lang A, Balmert LC, Weiss M et al (2022) Real world use of peanut component testing among children in the Chicago metropolitan area. Allergy Asthma Proc 43:226–233. 10.2500/aap.2022.43.220021 35524355 10.2500/aap.2022.43.220021 PMC10250144

[54] Duan L, Celik A, Hoang JA et al (2021) Basophil activation test shows high accuracy in the diagnosis of peanut and tree nut allergy: the markers of nut allergy study. Allergy 76:1800–1812. 10.1111/all.14695 33300157 10.1111/all.14695 PMC8608143

[55] Mustillo A, Paradis L, Des Roches A et al (2022) Specific IgE to total IgE ratio does not improve peanut diagnostic accuracy in adults. Int Arch Allergy Immunol 183:980–984. 10.1159/000524847 35675786 10.1159/000524847

[56] Kaur N, Mehr S, Katelaris C et al (2021) Added diagnostic value of peanut component testing: a cross-sectional study in Australian children. J Allergy Clin Immunol Pract 9:245-253.e4. 10.1016/j.jaip.2020.08.060 32942048 10.1016/j.jaip.2020.08.060

[57] Percival E, Bhatia R, Preece K et al (2020) Change in exhaled nitric oxide during peanut challenge is related to severity of reaction. Allergy, Asthma & Clinical Immunology 16:64. 10.1186/s13223-020-00464-8 10.1186/s13223-020-00464-8 PMC7386245 32834829

[58] Goldberg MR, Appel MY, Tobi K et al (2024) Validation of the NUT CRACKER diagnostic algorithm and prediction for Cashew and Pistachio co-allergy. J Allergy Clin Immunol 12:1273-1282.e5. 10.1016/j.jaip.2024.02.012 10.1016/j.jaip.2024.02.012 38382880

[59] Virkud YV, Chen Y-C, Stieb ES et al (2019) Analysis of oral food challenge outcomes in IgE-mediated food allergies to almond in a large cohort. J Allergy Clin Immunol Pract 7:2359-2368.e3. 10.1016/j.jaip.2019.03.049 30974209 10.1016/j.jaip.2019.03.049 PMC7380558

[60] Caballero LR, Treudler R, Delaroque N et al (2023) Peptide epitopes as biomarkers of soya sensitization in rBet v 1 immunotherapy of birch-related soya allergy. Clin Exp Allergy 53:316–326. 10.1111/cea.14224 36102274 10.1111/cea.14224

[61] Goldberg MR, Appel MY, Nega R et al (2021) A prospective validation of the NUT CRACKER diagnostic algorithm for walnut and pecan allergy with prediction of severity. J Allergy Clin Immunol Pract 9:265-274.e6. 10.1016/j.jaip.2020.09.041 33039644 10.1016/j.jaip.2020.09.041

[62] Faihs V, Kugler C, Bent RK et al (2023) Challenge-confirmed diagnosis restores quality of life in cofactor-dependent wheat allergy. Ann Allergy Asthma Immunol 131:494-500.e1. 10.1016/j.anai.2023.06.008 37315737 10.1016/j.anai.2023.06.008

[63] Valbuena T, Reche M, Marco G et al (2021) Storage proteins are driving pediatric hazelnut allergy in a lipid transfer protein-rich area. Foods 10:2463. 10.3390/foods10102463 34681512 10.3390/foods10102463 PMC8535272

[64] Datema MR, Eller E, Zwinderman AH et al (2019) Ratios of specific IgG4 over IgE antibodies do not improve prediction of peanut allergy nor of its severity compared to specific IgE alone. Clin Exp Allergy 49:216–226. 10.1111/cea.13286 30269403 10.1111/cea.13286 PMC7379576

[65] Lang A, Kubala S, Grieco MC et al (2023) Severe food allergy reactions are associated with α-tryptase. J Allergy Clin Immunol 152:933–939. 10.1016/j.jaci.2023.07.014 37558059 10.1016/j.jaci.2023.07.014 PMC10592152

[66] Cottel N, Saf S, Bourgoin-Heck M et al (2021) Two different composite markers predict severity and threshold dose in peanut allergy. J Allergy Clin Immunol Pract 9:275-282.e1. 10.1016/j.jaip.2020.09.043 33038591 10.1016/j.jaip.2020.09.043

[67] Alves PB, Pereira HP, Alves MP et al (2022) Predictors of anaphylaxis to peanut and tree nuts in a mediterranean population. Allergy Asthma Proc 43:533–542. 10.2500/aap.2022.43.220060 36335421 10.2500/aap.2022.43.220060

[68] Kubota K, Nagakura K-I, Itonaga T et al (2022) Macadamia nut-specific IgE levels for predicting anaphylaxis. Pediatr Allergy Immunol 33:e13852. 10.1111/pai.13852 36156824 10.1111/pai.13852

[69] Petek T, Lajhar M, Krašovec B et al (2023) Risk factors for anaphylaxis in children allergic to peanuts. Medicina (B Aires) 59:1037. 10.3390/medicina59061037 10.3390/medicina59061037 PMC10300811 37374241

[70] Li J-D, Du Z-R, Liu J et al (2020) Characteristics of pollen-related food allergy based on individual pollen allergy profiles in the Chinese population. World Allergy Organ J 13:100120. 10.1016/j.waojou.2020.100120 32435327 10.1016/j.waojou.2020.100120 PMC7229292

[71] Ando Y, Miyamoto M, Kato M et al (2020) *Pru p* 7 predicts severe reactions after ingestion of peach in Japanese children and adolescents. Int Arch Allergy Immunol 181:183–190. 10.1159/000504367 31822011 10.1159/000504367

[72] Deng S, Yin J (2019) Clinical utility of basophil activation test in diagnosis and predicting severity of mugwort pollen-related peach allergy. World Allergy Organ J 12:100043. 10.1016/j.waojou.2019.100043 31316713 10.1016/j.waojou.2019.100043 PMC6593310

[73] Asaumi T, Sato S, Yanagida N et al (2019) IgE-specific *Pru p* 4 negatively predicts systemic allergy reaction to peach among Japanese children. Allergol Int 68:546–548. 10.1016/j.alit.2019.05.005 31231007 10.1016/j.alit.2019.05.005

[74] Sampson HA, van Gerth Wijk R, Bindslev-Jensen C et al (2012) Standardizing double-blind, placebo-controlled oral food challenges: American Academy of Allergy, Asthma & Immunology-European Academy of Allergy and Clinical Immunology PRACTALL consensus report. J Allergy Clin Immunol 130:1260–1274. 10.1016/j.jaci.2012.10.017 23195525 10.1016/j.jaci.2012.10.017

[75] Ring J, Messmer K (1977) Incidence and severity of anaphylactoid reactions to colloid volume substitutes. Lancet Lond Engl 1:466–469. 10.1016/s0140-6736(77)91953-5 10.1016/s0140-6736(77)91953-5 65572

[76] Muraro A, Werfel T, Hoffmann-Sommergruber K et al (2014) EAACI food allergy and anaphylaxis guidelines: diagnosis and management of food allergy. Allergy 69:1008–1025. 10.1111/all.12429 24909706 10.1111/all.12429

[77] Datema MR, Lyons SA, Fernández-Rivas M et al (2021) Estimating the risk of severe peanut allergy using clinical background and IgE sensitization profiles. Front Allergy 2:670789. 10.3389/falgy.2021.670789 35386994 10.3389/falgy.2021.670789 PMC8974676

[78] Kallen EJJ, Revers A, Fernández-Rivas M et al (2023) A European-Japanese study on peach allergy: IgE to Pru p 7 associates with severity. Allergy 78:2497–2509. 10.1111/all.15783 37334557 10.1111/all.15783

[79] Al-Ahmad M, Jusufovic E, Arifhodzic N, Rodriguez-Bouza T (2022) Peanut component Ara h 1 and 2 sensitization in patients with food allergy in Kuwait. Int Arch Allergy Immunol 183:315–321. 10.1159/000519297 34700320 10.1159/000519297

[80] Urbani S, Aruanno A, Gasbarrini A et al (2022) Epinephrine auto-injector prescription and use: a retrospective analysis and clinical risk assessment of adult patients sensitized to lipid transfer protein. Nutrients 14:2706. 10.3390/nu14132706 35807887 10.3390/nu14132706 PMC9269022

[81] Elegbede CF, Papadopoulos A, Just J et al (2019) Gender, prick test size and rAra h 2 sIgE level may predict the eliciting dose in patients with peanut allergy: evidence from the Mirabel survey. Clin Exp Allergy 49:677–689. 10.1111/cea.13348 30689235 10.1111/cea.13348

[82] Suprun M, Kearney P, Hayward C et al (2022) Predicting probability of tolerating discrete amounts of peanut protein in allergic children using epitope-specific IgE antibody profiling. Allergy 77:3061–3069. 10.1111/all.15477 35960650 10.1111/all.15477 PMC10286745

[83] Berin MC, Agashe C, Burks AW et al (2022) Allergen-specific T cells and clinical features of food allergy: lessons from CoFAR immunotherapy cohorts. J Allergy Clin Immunol 149:1373-1382.e12. 10.1016/j.jaci.2021.09.029 34653515 10.1016/j.jaci.2021.09.029 PMC8995337

[84] Dreskin SC, Germinaro M, Reinhold D et al (2019) IgE binding to linear epitopes of Ara h 2 in peanut allergic preschool children undergoing oral immunotherapy. Pediatr Allergy Immunol 30:817–823. 10.1111/pai.13117 31437325 10.1111/pai.13117 PMC6906227

[85] Itonaga T, Yanagida N, Nagakura K-I et al (2024) Three-year prognosis after low-dose oral food challenge for children with wheat allergy. Allergol Int 73:416–421. 10.1016/j.alit.2024.01.004 38296769 10.1016/j.alit.2024.01.004

[86] Ruiter B, Smith NP, Monian B et al (2020) Expansion of the CD4 + effector T-cell repertoire characterizes peanut-allergic patients with heightened clinical sensitivity. J Allergy Clin Immunol 145:270–282. 10.1016/j.jaci.2019.09.033 31654649 10.1016/j.jaci.2019.09.033 PMC6949413

[87] Zhang L, Chun Y, Ho H-E et al (2022) Multiscale study of the oral and gut environments in children with high- and low-threshold peanut allergy. J Allergy Clin Immunol 150:714-720.e2. 10.1016/j.jaci.2022.04.026 35550149 10.1016/j.jaci.2022.04.026 PMC9463091

[88] Moraly T, de Pelletier Chambure D, Verdun S et al (2020) Oral immunotherapy for hazelnut allergy: a single-center retrospective study on 100 patients. The Journal of Allergy and Clinical Immunology: In Practice 8:704-709.e4. 10.1016/j.jaip.2019.10.045 31751759 10.1016/j.jaip.2019.10.045

[89] Jones SM, Kim EH, Nadeau KC et al (2022) Efficacy and safety of oral immunotherapy in children aged 1–3 years with peanut allergy (the Immune Tolerance Network IMPACT trial): a randomised placebo-controlled study. Lancet 399:359–371. 10.1016/S0140-6736(21)02390-4 35065784 10.1016/S0140-6736(21)02390-4 PMC9119642

[90] Tsai M, Mukai K, Chinthrajah RS et al (2020) Sustained successful peanut oral immunotherapy associated with low basophil activation and peanut-specific IgE. J Allergy Clin Immunol 145:885-896.e6. 10.1016/j.jaci.2019.10.038 31805311 10.1016/j.jaci.2019.10.038 PMC6957313

[91] Pourvali A, Arshi S, Nabavi M et al (2023) Sustained unresponsiveness development in wheat oral immunotherapy: predictive factors and flexible regimen in the maintenance phase. Eur Ann Allergy Clin Immunol 55:174–179. 10.23822/EurAnnACI.1764-1489.254 35620981 10.23822/EurAnnACI.1764-1489.254

[92] O’B Hourihane J, Beyer K, Abbas A et al (2020) Efficacy and safety of oral immunotherapy with AR101 in European children with a peanut allergy (ARTEMIS): a multicentre, double-blind, randomised, placebo-controlled phase 3 trial. Lancet Child Adolesc Health 4:728–739. 10.1016/S2352-4642(20)30234-0 32702315 10.1016/S2352-4642(20)30234-0

[93] Davis CM, Anagnostou A, Devaraj S et al (2022) Maximum dose food challenges reveal transient sustained unresponsiveness in peanut oral immunotherapy (POIMD study). J Allergy Clin Immunol Pract 10:566-576.e6. 10.1016/j.jaip.2021.10.074 34890827 10.1016/j.jaip.2021.10.074 PMC10404846

[94] Yee CSK, Albuhairi S, Noh E et al (2019) Long-term outcome of peanut oral immunotherapy facilitated initially by Omalizumab. J Allergy Clin Immunol Pract 7:451-461.e7. 10.1016/j.jaip.2018.09.015 30267889 10.1016/j.jaip.2018.09.015

[95] Rambo IM, Kronfel CM, Rivers AR et al (2023) IgE and IgG4 epitopes of the peanut allergens shift following oral immunotherapy. Front Allergy 4:1279290. 10.3389/falgy.2023.1279290 38093814 10.3389/falgy.2023.1279290 PMC10717846

[96] Suprun M, Lee ASE, Getts R et al (2025) Baseline epitope-specific IgE profiles are predictive of sustained unresponsiveness or high threshold 1-year post oral immunotherapy in the POISED trial. J Allergy Clin Immunol 155:923-931.e2. 10.1016/j.jaci.2024.10.017 39505279 10.1016/j.jaci.2024.10.017 PMC12893418

[97] Scurlock AM, Burks AW, Sicherer SH et al (2021) Epicutaneous immunotherapy for treatment of peanut allergy: follow-up from the Consortium for Food Allergy Research. J Allergy Clin Immunol 147:992-1003.e5. 10.1016/j.jaci.2020.11.027 33290772 10.1016/j.jaci.2020.11.027 PMC8612061

[98] Bastin M, Carr WW, Davis CM et al (2023) Immune response evolution in peanut epicutaneous immunotherapy for peanut-allergic children. Allergy 78:2467–2476. 10.1111/all.15709 36916639 10.1111/all.15709

[99] Fleischer DM, Chinthrajah S, Scurlock AM et al (2020) An evaluation of factors influencing response to epicutaneous immunotherapy for peanut allergy in the PEPITES trial. Allergy Asthma Proc 41:326–335. 10.2500/aap.2020.41.200047 32539908 10.2500/aap.2020.41.200047

[100] Shahali Y, Dadar M (2018) Plant food allergy: influence of chemicals on plant allergens. Food Chem Toxicol Int J Publ Br Ind Biol Res Assoc 115:365–374. 10.1016/j.fct.2018.03.032 10.1016/j.fct.2018.03.032 29580820

[101] Garcia-Blanca A, Aranda A, Blanca-Lopez N et al (2015) Influence of age on IgE response in peanut-allergic children and adolescents from the Mediterranean area. Pediatr Allergy Immunol 26:497–502. 10.1111/pai.12418 26046378 10.1111/pai.12418

[102] Masthoff LJ, van Hoffen E, de Reus A et al (2014) Hazelnut allergy differs between children and adults in frequency of severity, aetiology and relevance of diagnostic parameters. Clin Exp Allergy 44:1539–1545. 10.1111/cea.12440 25333730 10.1111/cea.12440

[103] Ballmer-Weber BK, Lidholm J, Fernández-Rivas M et al (2015) IgE recognition patterns in peanut allergy are age dependent: perspectives of the EuroPrevall study. Allergy 70:391–407. 10.1111/all.12574 25620497 10.1111/all.12574

[104] Zuidmeer L, Goldhahn K, Rona RJ et al (2008) The prevalence of plant food allergies: a systematic review. J Allergy Clin Immunol 121:1210-1218.e4. 10.1016/j.jaci.2008.02.019 18378288 10.1016/j.jaci.2008.02.019

[105] Shu S-A, Yuen AWT, Woo E et al (2019) Microbiota and food allergy. Clin Rev Allergy Immunol 57:83–97. 10.1007/s12016-018-8723-y 30564985 10.1007/s12016-018-8723-y

[106] Caminero A, Sánchez-Martínez E, Wulczynski M et al (2025) Potential role of microbes in antigen-based food sensitivities. Semin Immunol 79:101982. 10.1016/j.smim.2025.101982 40779882 10.1016/j.smim.2025.101982

[107] Sato S, Ebisawa M (2024) Precision allergy molecular diagnosis applications in food allergy. Curr Opin Allergy Clin Immunol 24:129–137. 10.1097/ACI.0000000000000977 38529801 10.1097/ACI.0000000000000977

[108] Al-Shaikhly T, Cox A, Nowak-Wegrzyn A et al (2024) An international Delphi consensus on the management of pollen-food allergy syndrome: a work group report of the AAAAI adverse reactions to foods committee. J Allergy Clin Immunol Pract 12:3242-3249.e1. 10.1016/j.jaip.2024.09.037 39488768 10.1016/j.jaip.2024.09.037 PMC11625607

[109] Roberts G, Ollert M, Aalberse R et al (2016) A new framework for the interpretation of IgE sensitization tests. Allergy 71:1540–1551. 10.1111/all.12939 27224838 10.1111/all.12939

[110] Lord SJ, Horvath AR, Sandberg S et al (2025) Is this test fit-for-purpose? Principles and a checklist for evaluating the clinical performance of a test in the new era of *in vitro* diagnostic (IVD) regulation. Crit Rev Clin Lab Sci 62(3):182–197. 10.1080/10408363.2025.2453148 39912349 10.1080/10408363.2025.2453148

