# Supplementary material for: Precision Medicine in Plant Food Allergy: a Systematic Review of Biomarkers Under a Clinical Approach
Source: Clin Rev Allergy Immunol. 2026 Feb 25;69(1):10. doi: 10.1007/s12016-026-09136-8 (PMC12935746; doi:10.1007/s12016-026-09136-8)
Supplement: Supplementary file 1 — Supplementary Material 1 [file 12016_2026_9136_MOESM1_ESM.docx]

**SUPPLEMENTARY TABLES**

**Supplementary Table 1.** Common and specific search codes for the five thematic sections. The search syntax was adapted to each database: *PubMed*, *Web of Sciences* and *Cochrane Library*.

| Search code |
| --- |
| 0. Common to all sections |
| ("plants, edible" OR ("plants" AND "edible") OR "edible plants" OR ("plant" AND "food") OR "plant food" OR "prunus persica" OR ("prunus" AND "persica") OR "prunus persica" OR "peach*" OR "arachis" OR "arachis" OR "peanut*" OR "triticum" OR "triticum" OR "wheat*" OR "nuts" OR "nut" OR "nuts" OR "actinidia" OR "actinidia" OR "kiwifruit*" OR "malus" OR "malus" OR "apple*" OR "lipid transfer protein" OR "LTP" OR "profilins" OR "profilin*" OR "PR-10") AND ("hypersensitivity" OR "hypersensitivity" OR "allergies" OR "allergy" OR "allergic" OR "allergy and immunology" OR ("allergy" AND "immunology") OR "allergy and immunology" OR "food hypersensitivity" OR ("food" AND "hypersensitivity") OR "food hypersensitivity" OR ("food" AND "allergy") OR "food allergy") AND ("biomarker*" OR "predict*" OR "omic*" OR "proteomic*" OR "transcriptomic*" OR "genomic*" OR "lipidomic*" OR "metabolomic*" OR "breathomic*" OR "-omic*" OR "glycomic*" OR "marker*" OR "precission" OR "endotyp*" OR "phenotype" OR "phenotyp*") AND ("human*" OR "patient*" OR "child*" OR "person*") |
| 1. Sensitization |
| ("sensitiz*" OR "atopic march" OR "allergic march" OR (("gene" OR "genet*" OR "polymorphism*" OR "SNP" OR "SNPs" OR "mutation*") AND "sensitiz*") OR (("pollution" OR "microplastic*" OR "diet*" OR "environment*" OR "habitat*" OR "processed food*" OR "emulsifier*" OR "additive*" OR "PM2.5" OR "tobacco" OR "smoke*" OR "fire" OR "diesel" OR "microbiota" OR "microbiome" OR "patho*" OR "infection*" OR "virus" OR "viral" OR "comorbidity" OR "epithelial barrier" OR "damage" OR "alarmin*" OR "eczema") AND "sensitiz*") AND ("predict**" OR "risk" OR "prognos*") |
| 2. Tolerance |
| ("sensitivity and specificity" OR ("sensitivity" AND "specificity") OR "sensitivity and specificity" OR "specificity" OR "specific*" OR "sensitivity") AND ("toler*") NOT ("intolerance" OR "intolerances") AND ("predict**" OR "risk" OR "prognos*") |
| 3. Severity |
| ("sever*" OR "mortality" OR "morbility" OR "death*") AND ("anaphy*" OR ("systemic" AND "reaction*") OR ("lethal" AND "reaction*")) AND ("predict*" OR "risk" OR "prognos*" OR "susceptibility") |
| 4. Threshold |
| ("threshold*" OR "reactive dose*") AND (("mouth" OR "mouth" OR "oral") OR "challenge*" OR "oral food challenge*" OR "OFC" OR "DBPCFC") |
| 5. Follow-up treatment |
| ("immunotherap*" OR "therap*" OR "treatment*" OR "OIT" OR "AIT" OR "SLIT" OR "SCIT" OR "EPIT") AND ("monitor*" OR "tracing" OR "check*" OR "track*" OR "sustained unresponsiveness") |

**Supplemental Table 2.** Risk-of-bias for non-randomized studies according to the Risk-Of-Bias In Non-randomized Studies of Interventions (ROBINS-I) tool. *Italics** indicates a change in the artificial intelligence (AI) evaluation based on human review.

| **Article (year)** | **Thematic section** | **Confounding** | **Selection of participants** | **Classification of exposures** | **Deviations** | **Missing data** | **Measurement of outcomes** | **Selection of result** | **Overall risk of bias** | **Reference** |
| --- | --- | --- | --- | --- | --- | --- | --- | --- | --- | --- |
| Lee-Sarwar *et al.* (2023) | Sensitization | Moderate | Low | Low | N/A | Low | Moderate | Low | **Moderate** | [1] |
| Wärnberg Gerdin *et al.* (2022) | Sensitization | Moderate | Low | Low | N/A | Low | *Low** | Low | **Moderate** | [2] |
| Alves *et al.* (2022) | Severity | Moderate | Moderate | Low | N/A | Low | Moderate | Low | **Moderate** | [3] |
| Ehlers *et al.* (2021) | Tolerance | *Low** | Low | Low | N/A | Low | Moderate | Low | ***Low**** | [4] |
| Tsilochristou *et al.* (2019) | Sensitivity | Moderate | Low | Low | N/A | Low | *Low** | Low | ***Low**** | [5] |
| Urbani *et al.* (2022) | Severity | Moderate | Low | Low | N/A | *Low** | *Low** | Low | **Low** | [6] |
| Ji *et al.* (2023) | Tolerance | *Low** | Low | Low | N/A | Moderate | Low | Low | **Moderate** | [7] |
| Ramírez Caballero *et al.* (2023) | Severity, Threshold | Moderate | Low | Low | N/A | Low | Moderate | Low | **Moderate** | [8] |
| Goleva *et al.* (2020) | Sensitization | Moderate | Low | Low | N/A | Low | Moderate | Low | **Moderate** | [9] |
| Moraly *et al.* (2020) | Follow-up treatment | Moderate | Low | Low | Low | Low | Moderate | Low | **Moderate** | [10] |
| Faihs *et al.* (2023) | Severity, Threshold | Moderate | Low | Low | N/A | Low | Moderate | Low | **Moderate** | [11] |
| Lee-Sarwar *et al.* (2019) | Sensitization | Moderate | Low | Low | N/A | Low | Moderate | Low | **Moderate** | [12] |
| Klueber *et al.* (2023) | Tolerance, Threshold | *Low** | Low | Low | N/A | Low | Moderate | Low | **Moderate** | [13] |
| Tedner *et al.* (2021) | Sensitization | Low | Low | Low | N/A | Moderate | Low | Low | **Low** | [14] |
| Ran *et al.* (2024) | Sensitization | Moderate | Low | Low | N/A | Moderate | *Low** | Low | **Moderate** | [15] |
| Berin *et al.* (2022) | Threshold, Follow-up treatment | Low | Low | Low | *Low** | Low | Low | Low | **Low** | [16] |
| Itonaga *et al.* (2024) | Threshold, Follow-up treatment | Moderate | Low | Low | *Moderate** | Moderate | Moderate | Low | **Moderate** | [17] |
| Ali Pourvali *et al.* (2023) | Follow-up treatment | Moderate | Low | Low | Low | Moderate | Moderate | Low | **Moderate** | [18] |
| Dreskin *et al.* (2019) | Threshold, Follow-up treatment | Moderate | Low | Low | N/A | Moderate | Moderate | Low | **Moderate** | [19] |
| Ruiter *et al.* (2020) | Threshold | Low | *Moderate** | Low | N/A | Low | Low | Low | ***Moderate**** | [20] |
| Lang *et al.* (2023) | Severity | Moderate | Moderate | Low | N/A | Low | Moderate | Low | **Moderate** | [21] |
| Petek *et al.* (2023) | Severity | Moderate | Moderate | Low | N/A | Low | Moderate | Low | **Moderate** | [22] |
| Davis *et al.* (2022) | Follow-up treatment | Moderate | Low | Low | Low | *Low** | Moderate | Low | **Moderate** | [23] |
| Zhang *et al.* (2022) | Threshold | Moderate | Low | Low | N/A | Moderate | Low | Low | **Moderate** | [24] |
| Yee *et al.* (2019) | Follow-up treatment | Moderate | Low | Low | Low | Moderate | Moderate | Low | **Moderate** | [25] |
| Rambo *et al.* (2023) | Follow-up treatment | Moderate | Low | Low | N/A | Moderate | Low | Low | **Moderate** | [26] |
| Suprun *et al.* (2024) | Follow-up treatment | Moderate | Low | Low | N/A | Moderate | Low | Low | **Moderate** | [27] |

**Supplemental Table 3.** Risk-of-bias for studies of diagnostic accuracy according to the Quality Assessment of Diagnostic Accuracy Studies (QUADAS-2) tool. *Italics** indicates a change in the artificial intelligence (AI) evaluation based on human review.

| **Article (year)** | **Thematic section** | **Patient selection** | **Index test** | **Reference standard** | **Flow and timing** | **Applicability concerns** | **Overall risk of bias** | **Reference** |
| --- | --- | --- | --- | --- | --- | --- | --- | --- |
| Santos *et al.* (2021) | Tolerance | Low | Low | Low | Low | Low | **Low** | [28] |
| Santos *et al.* (2020) | Tolerance, Severity, Threshold | Low | Low | Low | Low | Low | **Low** | [29] |
| Gur Cetinkaya *et al.* (2021) | Tolerance | Moderate | Low | Moderate | Moderate | Low | **Moderate** | [30] |
| Goldberg *et al.* (2024) | Severity, Threshold | Low | Low | Low | Low | Low | **Low** | [31] |
| Röntynen *et al.* (2022) | Tolerance, Severity, Threshold | Low | Low | *Low** | Low | Low | **Low** | [32] |
| Kaur *et al.* (2021) | Tolerance, Severity, Threshold | Moderate | Low | Moderate | Low | Low | **Moderate** | [33] |
| Lang *et al.* (2022) | Tolerance | Moderate | Low | Moderate | Moderate | Low | **Moderate** | [34] |
| Duan *et al.* (2021) | Tolerance | Low | Low | Low | Low | Low | **Low** | [35] |
| Virkud *et al.* (2019) | Severity | Moderate | Low | Moderate | Low | Low | **Moderate** | [36] |
| Inoue *et al.* (2020) | Tolerance | Moderate | Low | Moderate | Moderate | Low | **Moderate** | [37] |
| Ando *et al.* (2019) | Severity | Moderate | Low | Moderate | Low | Low | **Moderate** | [38] |
| Goldberg *et al.* (2020) | Severity, Threshold | Low | Low | Low | Low | Low | **Low** | [39] |
| Valbuena *et al.* (2021) | Severity | Moderate | Low | Moderate | Low | Low | **Moderate** | [40] |
| Deng *et al.* (2019) | Severity | Low | Low | Moderate | Low | Low | **Low** | [41] |
| Ruinemans-Koerts *et al.* (2022) | Tolerance, Threshold | Low | Low | Low | Low | Low | **Low** | [42] |
| Machnes-Maayan *et al.* (2022) | Tolerance | Moderate | Low | Moderate | Low | Low | **Moderate** | [43] |
| Percival *et al.* (2020) | Tolerance, Severity | Moderate | Low | Moderate | Low | *Moderate** | **Moderate** | [44] |
| Kansen *et al.* (2021) | Tolerance | Low | Low | Low | Low | Low | **Low** | [45] |
| Carrette *et al.* (2023) | Tolerance | *Low** | Low | Moderate | Low | Low | **Moderate** | [46] |
| Kidon *et al.* (2021) | Tolerance | Low | Low | *Moderate** | Low | Low | **Low** | [47] |
| Kubota *et al.* (2022) | Severity | Moderate | Low | Moderate | Low | Low | **Moderate** | [48] |
| Chua *et al.* (2021) | Tolerance | Moderate | Low | Moderate | Low | Low | **Moderate** | [49] |
| Ojaniemi *et al.* (2022) | Tolerance | Moderate | Low | Moderate | Low | *Moderate** | **Moderate** | [50] |
| Datema *et al.* (2019) | Severity | Low | Low | Low | Low | Low | **Low** | [51] |
| Mustillo *et al.* (2022) | Tolerance | Moderate | Low | Moderate | Low | Low | **Moderate** | [52] |
| Suprun *et al.* (2022) | Threshold | Low | Low | Low | Low | Low | **Low** | [53] |
| Fujisawa *et al.* (2021) | Tolerance | Moderate | Low | Moderate | Low | Low | **Moderate** | [54] |
| Kansen *et al.* (2021) | Tolerance | Low | Low | Moderate | Low | Low | **Low** | [55] |
| Cottel *et al.* (2021) | Severity, Threshold | Moderate | Low | Moderate | Low | Low | **Moderate** | [56] |

**Supplemental Table 4.** Risk-of-bias for cross-sectional studies according to the Appraisal tool for Cross-Sectional Studies (AXIS) gadget. *Italics** indicates a change in the artificial intelligence (AI) evaluation based on human review.

| **Article (year)** | **Thematic section** | **Clear aims and justification** | **Appropriate study design** | **Sample size and justification** | **Selection of participants** | **Measurement of validity and reliability** | **Risk of non-response bias** | **Statistical methods and reporting** | **Ethics and disclosures** | **Overall risk of bias** | **Reference** |
| --- | --- | --- | --- | --- | --- | --- | --- | --- | --- | --- | --- |
| Kallen *et al.* (2023) | Severity | Low | Low | Moderate | Low | *Moderate** | Moderate | Low | Low | **Moderate** | [57] |
| Lyons *et al.* (2021) | Severity | Low | Low | Moderate | Low | Low | Moderate | Low | Low | **Low** | [58] |
| Tran *et al.* (2024) | Sensitization | Low | Low | Moderate | Low | Moderate | Low | Low | Low | **Moderate** | [59] |
| Datema *et al.* (2021) | Severity | Low | Low | Low | Low | Low | Low | Low | Low | **Low** | [60] |
| Al-Ahmad *et al.* (2022) | Severity | Low | Low | Moderate | Moderate | Low | Low | Low | Low | **Moderate** | [61] |
| Elegbede *et al.* (2019) | Threshold | Low | Low | Moderate | Moderate | Low | Low | Low | Low | **Moderate** | [62] |
| Lee *et al.* (2021) | Tolerance | Low | Low | Moderate | Moderate | Low | Moderate | Low | Low | **Moderate** | [63] |
| Asaumi *et al.* (2019) | Severity | Low | Low | Moderate | Moderate | Low | Moderate | Low | Low | **Moderate** | [64] |
| Li *et al.* (2020) | Severity | Low | Low | Moderate | Moderate | Moderate | Low | Low | Low | **Moderate** | [65] |

**Supplemental Table 5.** Risk-of-bias for studies of randomized trials according to the Cochrane tool for assessing risk-of-bias in randomized trials (Rob2) gadget. *Italics** indicates a change in the artificial intelligence (AI) evaluation based on human review.

| **Article (year)** | **Thematic section** | **Randomization process** | **Deviations from intended interventions** | **Missing outcome data** | **Measurement of the outcome** | **Selection of reported result** | **Overall risk of bias** | **Reference** |
| --- | --- | --- | --- | --- | --- | --- | --- | --- |
| Jones *et al.* (2022) | Follow-up treatment | Low | Low | Low | Low | Low | **Low** | [66] |
| Tsai *et al.* (2020) | Follow-up treatment | Low | Low | Low | Low | Low | **Low** | [67] |
| Nadeau *et al.* (2023) | Follow-up treatment | Low | Low | Low | Low | Low | **Low** | [68] |
| Bastin *et al.* (2023) | Follow-up treatment | Low | Low | Low | Low | Low | **Low** | [69] |
| O’B Hourihane *et al.* (2020) | Follow-up treatment | Low | Low | Low | Low | Low | **Low** | [70] |
| Fleischer *et al.* (2020) | Follow-up treatment | Low | Low | Low | Low | Low | **Low** | [71] |

**Supplemental Table 6.** Determination of the certainty of evidence from all studies using the Grading of Recommendations, Assessment, Development, and Evaluation (GRADE) system. *Italics** indicates a change in the artificial intelligence (AI) evaluation based on human review.

| **Article (year)** | **Thematic section** | **Risk of Bias** | **Inconsistency** | **Imprecision** | **Indirect Evidence** | **Publication Bias** | **Overall Certainty Level** | **Reference** |
| --- | --- | --- | --- | --- | --- | --- | --- | --- |
| Lee-Sarwar *et al.* (2023) | Sensitization | Moderate | Yes | *Moderate** | Some | Possible | **Low** | [1] |
| Wärnberg Gerdin *et al.* (2022) | Sensitization | Low | No | Moderate | No | *Unclear** | **Moderate** | [2] |
| Alves *et al.* (2022) | Severity | Moderate | Yes | Moderate | Some | Possible | **Low** | [3] |
| Kallen *et al.* (2023) | Severity | Moderate | Yes | Moderate | *No** | Possible | ***Moderate**** | [57] |
| Ehlers *et al.* (2021) | Tolerance | Low | N/A | High | High | *Possible** | **Low** | [4] |
| Santos *et al.* (2021) | Tolerance | Moderate | Yes | Moderate | *No** | Possible | ***Moderate**** | [28] |
| Santos *et al.* (2020) | Tolerance, Severity, Threshold | Low | No | Low | No | Unlikely | **High** | [29] |
| Gur Cetinkaya *et al.* (2021) | Tolerance | Moderate | No | Moderate | Some | Possible | **Moderate** | [30] |
| Goldberg *et al.* (2024) | Severity, Threshold | Low | No | Low | No | Unlikely | **High** | [31] |
| Tsilochristou *et al.* (2019) | Sensitivity | Moderate | No | Moderate | *No** | Possible | ***High**** | [5] |
| Röntynen *et al.* (2022) | Tolerance, Severity, Threshold | Moderate | No | Moderate | *No** | Possible | **Moderate** | [32] |
| Kaur *et al.* (2021) | Tolerance, Severity, Threshold | Moderate | No | Moderate | No | Possible | **Moderate** | [33] |
| Lang *et al.* (2022) | Tolerance | Moderate | No | *Low** | No | *No** | **Moderate** | [34] |
| Lyons *et al.* (2021) | Severity | Moderate | No | Moderate | Some | Possible | **Moderate** | [58] |
| Duan *et al.* (2021) | Tolerance | Moderate | No | Moderate | No | *No** | **Moderate** | [35] |
| Urbani *et al.* (2022) | Severity | Moderate | No | Moderate | *No** | Possible | **Moderate** | [6] |
| Virkud *et al.* (2019) | Severity | Moderate | No | Moderate | No | Possible | **Moderate** | [36] |
| Ji *et al.* (2023) | Tolerance | Low | No | *Low** | No | Unlikely | ***High**** | [7] |
| Tran *et al.* (2024) | Sensitization | Moderate | No | Moderate | Some | Possible | **Moderate** | [59] |
| Inoue *et al.* (2020) | Tolerance | Moderate | No | Moderate | No | Possible | **Moderate** | [37] |
| Ramírez Caballero *et al.* (2023) | Severity, Threshold | Moderate | N/A | Moderate | High | *Unlikely** | **Moderate** | [8] |
| Goleva *et al.* (2020) | Sensitization | Moderate | No | Moderate | No | Unlikely | **Moderate** | [9] |
| Ando *et al.* (2019) | Severity | Moderate | No | Moderate | No | Possible | **Moderate** | [38] |
| Moraly *et al.* (2020) | Follow-up treatment | Moderate | No | Moderate | No | Possible | **Moderate** | [10] |
| Goldberg *et al.* (2020) | Severity, Threshold | Low | No | Moderate | No | Unlikely | **Moderate** | [39] |
| Faihs *et al.* (2023) | Severity, Threshold | Moderate | No | Moderate | Some | Possible | **Moderate** | [11] |
| Valbuena *et al.* (2021) | Severity | Moderate | No | Moderate | No | Possible | **Moderate** | [40] |
| Deng *et al.* (2019) | Severity | Moderate | No | *High** | Some | Possible | **Moderate** | [41] |
| Ruinemans-Koerts *et al.* (2022) | Tolerance, Threshold | Moderate | No | Moderate | No | Possible | **Moderate** | [42] |
| Machnes-Maayan *et al.* (2022) | Tolerance | Moderate | No | Moderate | Some | Possible | **Moderate** | [43] |
| Lee-Sarwar *et al.* (2019) | Sensitization | Moderate | No | Moderate | Some | Possible | **Moderate** | [12] |
| Percival *et al.* (2020) | Tolerance, Severity | Moderate | No | Moderate | Some | Possible | **Moderate** | [44] |
| Kansen *et al.* (2021) | Tolerance | *Low** | No | *Low** | No | *No** | ***High**** | [45] |
| Carrette *et al.* (2023) | Tolerance | Moderate | No | Moderate | No | Possible | **Moderate** | [46] |
| Datema *et al.* (2021) | Severity | Moderate | No | Moderate | Some | Possible | **Moderate** | [60] |
| Al-Ahmad *et al.* (2022) | Severity | Moderate | No | Moderate | Some | Possible | **Moderate** | [61] |
| Kidon *et al.* (2021) | Tolerance | Moderate | No | Moderate | No | Possible | **Moderate** | [47] |
| Kubota *et al.* (2022) | Severity | Moderate | No | Moderate | No | Possible | **Moderate** | [48] |
| Chua *et al.* (2021) | Tolerance | Moderate | No | Moderate | No | Possible | **Moderate** | [49] |
| Klueber *et al.* (2023) | Tolerance, Threshold | Moderate | No | Moderate | No | Unlikely | **Moderate** | [13] |
| Elegbede *et al.* (2019) | Threshold | Moderate | No | Moderate | No | Possible | **Moderate** | [62] |
| Ojaniemi *et al.* (2022) | Tolerance | Moderate | No | Moderate | Some | Possible | **Moderate** | [50] |
| Tedner *et al.* (2021) | Sensitization | Moderate | No | Moderate | *No** | Possible | **Moderate** | [14] |
| Datema *et al.* (2019) | Severity | Moderate | No | Moderate | No | Possible | **Moderate** | [51] |
| Ran *et al.* (2024) | Sensitization | Moderate | No | Moderate | *No** | Possible | **Moderate** | [15] |
| Jones *et al.* (2022) | Follow-up treatment | Low | No | Moderate | No | Unlikely | **High** | [66] |
| Berin *et al.* (2022) | Thershold, Follow-up treatment | Moderate | No | Moderate | No | Possible | **Moderate** | [16] |
| Mustillo *et al.* (2022) | Tolerance | Moderate | No | Moderate | No | Possible | **Moderate** | [52] |
| Tsai *et al.* (2020) | Follow-up treatment | Moderate | No | Moderate | No | Possible | **Moderate** | [67] |
| Nadeau *et al.* (2023) | Follow-up treatment | Low | No | Moderate | No | Unlikely | **High** | [68] |
| Suprun *et al.* (2022) | Threshold | Moderate | No | Moderate | No | *Unlikely** | **Moderate** | [53] |
| Itonaga *et al.* (2024) | Threshold, Follow-up treatment | Moderate | No | Moderate | Some | Possible | **Moderate** | [17] |
| Ali Pourvali *et al.* (2023) | Follow-up treatment | Moderate | No | Moderate | Some | Possible | **Moderate** | [18] |
| Lee *et al.* (2021) | Tolerance | Moderate | No | Moderate | Some | Possible | **Moderate** | [63] |
| Dreskin *et al.* (2019) | Thershold, Follow-up treatment | Moderate | No | Moderate | No | Possible | **Moderate** | [19] |
| Fujisawa *et al.* (2021) | Tolerance | Moderate | No | Moderate | No | Possible | **Moderate** | [54] |
| Ruiter *et al.* (2020) | Thershold | Moderate | No | Moderate | *Some** | Possible | **Moderate** | [20] |
| Kansen *et al.* (2021) | Tolerance | Moderate | No | Moderate | No | Possible | **Moderate** | [55] |
| Bastin *et al.* (2023) | Follow-up treatment | Low | No | Moderate | No | Unlikely | **Moderate** | [69] |
| Lang *et al.* (2023) | Severity | Moderate | No | Moderate | No | Possible | **Moderate** | [21] |
| Petek *et al.* (2023) | Severity | Moderate | No | Moderate | Some | Possible | **Moderate** | [22] |
| O’B Hourihane *et al.* (2020) | Follow-up treatment | Low | No | Moderate | No | Unlikely | **High** | [70] |
| Asaumi *et al.* (2019) | Severity | Moderate | No | Moderate | Some | Possible | **Moderate** | [64] |
| Cottel *et al.* (2021) | Severity, Threshold | Moderate | No | Moderate | No | Possible | **Moderate** | [56] |
| Davis *et al.* (2022) | Follow-up treatment | Moderate | No | Moderate | No | Possible | **Moderate** | [23] |
| Zhang *et al.* (2022) | Threshold | Moderate | No | Moderate | No | Possible | **Moderate** | [24] |
| Li *et al.* (2020) | Severity | Moderate | Yes | High | Some | Possible | **Low** | [65] |
| Fleischer *et al.* (2020) | Follow-up treatment | Low | No | Moderate | No | Unlikely | **Moderate** | [71] |
| Yee *et al.* (2019) | Follow-up treatment | Moderate | No | Moderate | No | Possible | **Moderate** | [25] |
| Rambo *et al.* (2023) | Follow-up treatment | Moderate | No | Moderate | No | Possible | **Moderate** | [26] |
| Suprun *et al.* (2024) | Follow-up treatment | Moderate | No | Moderate | No | Possible | **Moderate** | [27] |

**References**

1. Lee-Sarwar KA, Chen Y-C, Lasky-Su J, et al (2023) Early-life fecal metabolomics of food allergy. Allergy 78:512–521. https://doi.org/10.1111/all.15602

2. Wärnberg Gerdin S, Lie A, Asarnoj A, et al (2022) Impaired skin barrier and allergic sensitization in early infancy. Allergy 77:1464–1476. https://doi.org/10.1111/all.15170

3. Alves PB, Pereira HP, Alves MP, et al (2022) Predictors of anaphylaxis to peanut and tree nuts in a Mediterranean population. Allergy Asthma Proc 43:533–542. https://doi.org/10.2500/aap.2022.43.220060

4. Ehlers AM, den Hartog Jager CF, Knulst AC, Otten HG (2021) Distinction between peanut allergy and tolerance by characterization of B cell receptor repertoires. Allergy 76:2753–2764. https://doi.org/10.1111/all.14897

5. Tsilochristou O, du Toit G, Sayre PH, et al (2019) Association of Staphylococcus aureus colonization with food allergy occurs independently of eczema severity. J Allergy Clin Immunol 144:494–503. https://doi.org/10.1016/j.jaci.2019.04.025

6. Urbani S, Aruanno A, Gasbarrini A, et al (2022) Epinephrine Auto-Injector Prescription and Use: A Retrospective Analysis and Clinical Risk Assessment of Adult Patients Sensitized to Lipid Transfer Protein. Nutrients 14:2706. https://doi.org/10.3390/nu14132706

7. Ji C, Huang Y, Yeung LH, et al (2023) Ara h 2-Specific IgE Presence Rather Than Its Function Is the Best Predictor of Mast Cell Activation in Children. J Allergy Clin Immunol Pract 11:1154-1161.e3. https://doi.org/10.1016/j.jaip.2022.12.026

8. Caballero LR, Treudler R, Delaroque N, et al (2023) Peptide epitopes as biomarkers of soya sensitization in rBet v 1 immunotherapy of birch-related soya allergy. Clin Exp Allergy J Br Soc Allergy Clin Immunol 53:316–326. https://doi.org/10.1111/cea.14224

9. Goleva E, Calatroni A, LeBeau P, et al (2020) Skin tape proteomics identifies pathways associated with transepidermal water loss and allergen polysensitization in atopic dermatitis. J Allergy Clin Immunol 146:1367–1378. https://doi.org/10.1016/j.jaci.2020.04.022

10. Moraly T, Pelletier de Chambure D, Verdun S, et al (2020) Oral Immunotherapy for Hazelnut Allergy: A Single-Center Retrospective Study on 100 Patients. J Allergy Clin Immunol Pract 8:704-709.e4. https://doi.org/10.1016/j.jaip.2019.10.045

11. Faihs V, Kugler C, Bent RK, et al (2023) Challenge-confirmed diagnosis restores quality of life in cofactor-dependent wheat allergy. Ann Allergy Asthma Immunol Off Publ Am Coll Allergy Asthma Immunol 131:494-500.e1. https://doi.org/10.1016/j.anai.2023.06.008

12. Lee-Sarwar K, Kelly RS, Lasky-Su J, et al (2019) Dietary and Plasma Polyunsaturated Fatty Acids Are Inversely Associated with Asthma and Atopy in Early Childhood. J Allergy Clin Immunol Pract 7:529-538.e8. https://doi.org/10.1016/j.jaip.2018.07.039

13. Klueber J, Czolk R, Codreanu-Morel F, et al (2023) High-dimensional immune profiles correlate with phenotypes of peanut allergy during food-allergic reactions. Allergy 78:1020–1035. https://doi.org/10.1111/all.15408

14. Tedner SG, Söderhäll C, Konradsen JR, et al (2021) Extract and molecular-based early infant sensitization and associated factors-A PreventADALL study. Allergy 76:2730–2739. https://doi.org/10.1111/all.14805

15. Ran Z, Wang B, Zhang S-Y (2024) Associations of exposure to metals with total and allergen-specific IgE: An NHANES analysis (2005-2006). Sci Total Environ 906:167385. https://doi.org/10.1016/j.scitotenv.2023.167385

16. Berin MC, Agashe C, Burks AW, et al (2022) Allergen-specific T cells and clinical features of food allergy: Lessons from CoFAR immunotherapy cohorts. J Allergy Clin Immunol 149:1373-1382.e12. https://doi.org/10.1016/j.jaci.2021.09.029

17. Itonaga T, Yanagida N, Nagakura K-I, et al (2024) Three-year prognosis after low-dose oral food challenge for children with wheat allergy. Allergol Int Off J Jpn Soc Allergol 73:416–421. https://doi.org/10.1016/j.alit.2024.01.004

18. Pourvali A, Arshi S, Nabavi M, et al (2023) Sustained unresponsiveness development in wheat oral immunotherapy: predictive factors and flexible regimen in the maintenance phase. Eur Ann Allergy Clin Immunol 55:174–179. https://doi.org/10.23822/EurAnnACI.1764-1489.254

19. Dreskin SC, Germinaro M, Reinhold D, et al (2019) IgE binding to linear epitopes of Ara h 2 in peanut allergic preschool children undergoing oral Immunotherapy. Pediatr Allergy Immunol Off Publ Eur Soc Pediatr Allergy Immunol 30:817–823. https://doi.org/10.1111/pai.13117

20. Ruiter B, Smith NP, Monian B, et al (2020) Expansion of the CD4+ effector T-cell repertoire characterizes peanut-allergic patients with heightened clinical sensitivity. J Allergy Clin Immunol 145:270–282. https://doi.org/10.1016/j.jaci.2019.09.033

21. Lang A, Kubala S, Grieco MC, et al (2023) Severe food allergy reactions are associated with α-tryptase. J Allergy Clin Immunol 152:933–939. https://doi.org/10.1016/j.jaci.2023.07.014

22. Petek T, Lajhar M, Krašovec B, et al (2023) Risk Factors for Anaphylaxis in Children Allergic to Peanuts. Med Kaunas Lith 59:1037. https://doi.org/10.3390/medicina59061037

23. Davis CM, Anagnostou A, Devaraj S, et al (2022) Maximum Dose Food Challenges Reveal Transient Sustained Unresponsiveness in Peanut Oral Immunotherapy (POIMD Study). J Allergy Clin Immunol Pract 10:566-576.e6. https://doi.org/10.1016/j.jaip.2021.10.074

24. Zhang L, Chun Y, Ho H-E, et al (2022) Multiscale study of the oral and gut environments in children with high- and low-threshold peanut allergy. J Allergy Clin Immunol 150:714-720.e2. https://doi.org/10.1016/j.jaci.2022.04.026

25. Yee CSK, Albuhairi S, Noh E, et al (2019) Long-Term Outcome of Peanut Oral Immunotherapy Facilitated Initially by Omalizumab. J Allergy Clin Immunol Pract 7:451-461.e7. https://doi.org/10.1016/j.jaip.2018.09.015

26. Rambo IM, Kronfel CM, Rivers AR, et al (2023) IgE and IgG4 epitopes of the peanut allergens shift following oral immunotherapy. Front Allergy 4:1279290. https://doi.org/10.3389/falgy.2023.1279290

27. Suprun M, Lee ASE, Getts R, et al (2025) Baseline epitope-specific IgE profiles are predictive of sustained unresponsiveness or high threshold 1-year post oral immunotherapy in the POISED trial. J Allergy Clin Immunol 155:923-931.e2. https://doi.org/10.1016/j.jaci.2024.10.017

28. Santos AF, Bergmann M, Brough HA, et al (2021) Basophil Activation Test Reduces Oral Food Challenges to Nuts and Sesame. J Allergy Clin Immunol Pract 9:2016-2027.e6. https://doi.org/10.1016/j.jaip.2020.12.039

29. Santos AF, Du Toit G, O’Rourke C, et al (2020) Biomarkers of severity and threshold of allergic reactions during oral peanut challenges. J Allergy Clin Immunol 146:344–355. https://doi.org/10.1016/j.jaci.2020.03.035

30. Cetinkaya PG, Karaguzel D, Esenboğa S, et al (2021) Pistachio and cashew nut allergy in childhood: Predictive factors towards development of a decision tree. Asian Pac J Allergy Immunol 39:53–61. https://doi.org/10.12932/AP-281018-0429

31. Goldberg MR, Appel MY, Tobi K, et al (2024) Validation of the NUT CRACKER Diagnostic Algorithm and Prediction for Cashew and Pistachio Co-Allergy. J Allergy Clin Immunol Pract 12:1273-1282.e5. https://doi.org/10.1016/j.jaip.2024.02.012

32. Röntynen P, Kukkonen K, Savinko T, Mäkelä MJ (2022) Optimizing tools for evaluating challenge outcomes in children with cashew nut allergy. Ann Allergy Asthma Immunol Off Publ Am Coll Allergy Asthma Immunol 128:270–278. https://doi.org/10.1016/j.anai.2021.12.006

33. Kaur N, Mehr S, Katelaris C, et al (2021) Added Diagnostic Value of Peanut Component Testing: A Cross-Sectional Study in Australian Children. J Allergy Clin Immunol Pract 9:245-253.e4. https://doi.org/10.1016/j.jaip.2020.08.060

34. Lang A, Balmert LC, Weiss M, et al (2022) Real world use of peanut component testing among children in the Chicago metropolitan area. Allergy Asthma Proc 43:226–233. https://doi.org/10.2500/aap.2022.43.220021

35. Duan L, Celik A, Hoang JA, et al (2021) Basophil activation test shows high accuracy in the diagnosis of peanut and tree nut allergy: The Markers of Nut Allergy Study. Allergy 76:1800–1812. https://doi.org/10.1111/all.14695

36. Virkud YV, Chen Y-C, Stieb ES, et al (2019) Analysis of Oral Food Challenge Outcomes in IgE-Mediated Food Allergies to Almond in a Large Cohort. J Allergy Clin Immunol Pract 7:2359-2368.e3. https://doi.org/10.1016/j.jaip.2019.03.049

37. Inoue Y, Sato S, Takahashi K, et al (2020) Component-resolved diagnostics can be useful for identifying hazelnut allergy in Japanese children. Allergol Int Off J Jpn Soc Allergol 69:239–245. https://doi.org/10.1016/j.alit.2019.10.001

38. Ando Y, Miyamoto M, Kato M, et al (2020) Pru p 7 Predicts Severe Reactions after Ingestion of Peach in Japanese Children and Adolescents. Int Arch Allergy Immunol 181:183–190. https://doi.org/10.1159/000504367

39. Goldberg MR, Appel MY, Nega R, et al (2021) A Prospective Validation of the NUT CRACKER Diagnostic Algorithm for Walnut and Pecan Allergy with Prediction of Severity. J Allergy Clin Immunol Pract 9:265-274.e6. https://doi.org/10.1016/j.jaip.2020.09.041

40. Valbuena T, Reche M, Marco G, et al (2021) Storage Proteins Are Driving Pediatric Hazelnut Allergy in a Lipid Transfer Protein-Rich Area. Foods Basel Switz 10:2463. https://doi.org/10.3390/foods10102463

41. Deng S, Yin J (2019) Clinical utility of basophil activation test in diagnosis and predicting severity of mugwort pollen-related peach allergy. World Allergy Organ J 12:100043. https://doi.org/10.1016/j.waojou.2019.100043

42. Ruinemans-Koerts J, Brouwer ML, Schmidt-Hieltjes Y, et al (2022) The Indirect Basophil Activation Test Is a Safe, Reliable, and Accessible Tool to Diagnose a Peanut Allergy in Children. J Allergy Clin Immunol Pract 10:1305-1311.e3. https://doi.org/10.1016/j.jaip.2021.12.040

43. Machnes-Maayan D, Yahia SH, Frizinsky S, et al (2022) A clinical pathway for the diagnosis of sesame allergy in children. World Allergy Organ J 15:100713. https://doi.org/10.1016/j.waojou.2022.100713

44. Percival E, Bhatia R, Preece K, et al (2020) Change in exhaled nitric oxide during peanut challenge is related to severity of reaction. Allergy Asthma Clin Immunol Off J Can Soc Allergy Clin Immunol 16:64. https://doi.org/10.1186/s13223-020-00464-8

45. Kansen HM, van Erp FC, Knulst AC, et al (2021) Accurate Prediction of Peanut Allergy in One-Third of Adults Using a Validated Ara h 2 Cutoff. J Allergy Clin Immunol Pract 9:1667-1674.e3. https://doi.org/10.1016/j.jaip.2020.11.024

46. Carrette M, Couderc L, Bubenheim M, et al (2023) The combination of Ara h 2-sIgE and basophil activation test could be an alternative to oral food challenge in cases of suspected peanut allergy. Pediatr Allergy Immunol Off Publ Eur Soc Pediatr Allergy Immunol 34:e14007. https://doi.org/10.1111/pai.14007

47. Kidon MI, Yahia SH, Machnes-Maayan D, et al (2021) Diagnosis of Peanut Allergy in Preschool Children: The Impact of Skin Testing With a Novel Composition of Peanuts. Front Pediatr 9:739224. https://doi.org/10.3389/fped.2021.739224

48. Kubota K, Nagakura K-I, Itonaga T, et al (2022) Macadamia nut-specific IgE levels for predicting anaphylaxis. Pediatr Allergy Immunol Off Publ Eur Soc Pediatr Allergy Immunol 33:e13852. https://doi.org/10.1111/pai.13852

49. Chua GT, Chong PC, Au EY, et al (2021) Skin prick testing a better predictor than blood testing for the diagnosis of peanut allergy in Chinese children. Asian Pac J Allergy Immunol 39:241–248. https://doi.org/10.12932/AP-110319-0519

50. Ojaniemi I, Salmivesi S, Tikkakoski A, et al (2022) Are peanut oral food challenges still useful? An evaluation of children with suspected peanut allergy, sensitization to Ara h 2 and controlled asthma. Allergy Asthma Clin Immunol Off J Can Soc Allergy Clin Immunol 18:100. https://doi.org/10.1186/s13223-022-00743-6

51. Datema MR, Eller E, Zwinderman AH, et al (2019) Ratios of specific IgG4 over IgE antibodies do not improve prediction of peanut allergy nor of its severity compared to specific IgE alone. Clin Exp Allergy J Br Soc Allergy Clin Immunol 49:216–226. https://doi.org/10.1111/cea.13286

52. Mustillo A, Paradis L, Des Roches A, et al (2022) Specific IgE to Total IgE Ratio Does Not Improve Peanut Diagnostic Accuracy in Adults. Int Arch Allergy Immunol 183:980–984. https://doi.org/10.1159/000524847

53. Suprun M, Kearney P, Hayward C, et al (2022) Predicting probability of tolerating discrete amounts of peanut protein in allergic children using epitope-specific IgE antibody profiling. Allergy 77:3061–3069. https://doi.org/10.1111/all.15477

54. Al Hawi Y, Nagao M, Furuya K, et al (2021) Agreement Between Predictive, Allergen-Specific IgE Values Assessed by ImmunoCAP and IMMULITE 2000 3gAllergy^TM^ Assay Systems for Milk and Wheat Allergies. Allergy Asthma Immunol Res 13:141–153. https://doi.org/10.4168/aair.2021.13.1.141

55. Kansen HM, van Erp FC, Meijer Y, et al (2021) Diagnostic accuracy of Ara h 2 for detecting peanut allergy in children. Clin Exp Allergy J Br Soc Allergy Clin Immunol 51:1069–1079. https://doi.org/10.1111/cea.13987

56. Cottel N, Saf S, Bourgoin-Heck M, et al (2021) Two Different Composite Markers Predict Severity and Threshold Dose in Peanut Allergy. J Allergy Clin Immunol Pract 9:275-282.e1. https://doi.org/10.1016/j.jaip.2020.09.043

57. Kallen EJJ, Revers A, Fernández-Rivas M, et al (2023) A European-Japanese study on peach allergy: IgE to Pru p 7 associates with severity. Allergy 78:2497–2509. https://doi.org/10.1111/all.15783

58. Lyons SA, Datema MR, Le T-M, et al (2021) Walnut Allergy Across Europe: Distribution of Allergen Sensitization Patterns and Prediction of Severity. J Allergy Clin Immunol Pract 9:225-235.e10. https://doi.org/10.1016/j.jaip.2020.08.051

59. Tran NLH, Ly NTM, Trinh HKT, et al (2024) Prediction of Food Sensitization in Children with Atopic Dermatitis Based on Disease Severity and Epidermal Layer Impairment. Int Arch Allergy Immunol 185:43–55. https://doi.org/10.1159/000533492

60. Datema MR, Lyons SA, Fernández-Rivas M, et al (2021) Estimating the Risk of Severe Peanut Allergy Using Clinical Background and IgE Sensitization Profiles. Front Allergy 2:670789. https://doi.org/10.3389/falgy.2021.670789

61. Al-Ahmad M, Jusufovic E, Arifhodzic N, Rodriguez-Bouza T (2022) Peanut Component Ara h 1 and 2 Sensitization in Patients with Food Allergy in Kuwait. Int Arch Allergy Immunol 183:315–321. https://doi.org/10.1159/000519297

62. Elegbede CF, Papadopoulos A, Just J, et al (2019) Gender, prick test size and rAra h 2 sIgE level may predict the eliciting dose in patients with peanut allergy: Evidence from the Mirabel survey. Clin Exp Allergy J Br Soc Allergy Clin Immunol 49:677–689. https://doi.org/10.1111/cea.13348

63. Lee J, Jeong K, Jeon S-A, Lee S (2021) Component resolved diagnosis of walnut allergy in young children: Jug r 1 as a major walnut allergen. Asian Pac J Allergy Immunol 39:190–196. https://doi.org/10.12932/AP-161118-0443

64. Asaumi T, Sato S, Yanagida N, et al (2019) IgE-specific Pru p 4 negatively predicts systemic allergy reaction to peach among Japanese children. Allergol Int Off J Jpn Soc Allergol 68:546–548. https://doi.org/10.1016/j.alit.2019.05.005

65. Li J-D, Du Z-R, Liu J, et al (2020) Characteristics of pollen-related food allergy based on individual pollen allergy profiles in the Chinese population. World Allergy Organ J 13:100120. https://doi.org/10.1016/j.waojou.2020.100120

66. Jones SM, Kim EH, Nadeau KC, et al (2022) Efficacy and safety of oral immunotherapy in children aged 1-3 years with peanut allergy (the Immune Tolerance Network IMPACT trial): a randomised placebo-controlled study. Lancet Lond Engl 399:359–371. https://doi.org/10.1016/S0140-6736(21)02390-4

67. Tsai M, Mukai K, Chinthrajah RS, et al (2020) Sustained successful peanut oral immunotherapy associated with low basophil activation and peanut-specific IgE. J Allergy Clin Immunol 145:885-896.e6. https://doi.org/10.1016/j.jaci.2019.10.038

68. Scurlock AM, Burks AW, Sicherer SH, et al (2021) Epicutaneous immunotherapy for treatment of peanut allergy: Follow-up from the Consortium for Food Allergy Research. J Allergy Clin Immunol 147:992-1003.e5. https://doi.org/10.1016/j.jaci.2020.11.027

69. Bastin M, Carr WW, Davis CM, et al (2023) Immune response evolution in peanut epicutaneous immunotherapy for peanut-allergic children. Allergy 78:2467–2476. https://doi.org/10.1111/all.15709

70. O’B Hourihane J, Beyer K, Abbas A, et al (2020) Efficacy and safety of oral immunotherapy with AR101 in European children with a peanut allergy (ARTEMIS): a multicentre, double-blind, randomised, placebo-controlled phase 3 trial. Lancet Child Adolesc Health 4:728–739. https://doi.org/10.1016/S2352-4642(20)30234-0

71. Fleischer DM, Chinthrajah S, Scurlock AM, et al (2020) An evaluation of factors influencing response to epicutaneous immunotherapy for peanut allergy in the PEPITES trial. Allergy Asthma Proc 41:326–335. https://doi.org/10.2500/aap.2020.41.200047
